# Single-Cell Transcriptome Atlas of Newcastle Disease Virus in Chickens Both *In Vitro* and *In Vivo*

**DOI:** 10.1128/spectrum.05121-22

**Published:** 2023-05-16

**Authors:** Weiwei Liu, Zejun Xu, Yafeng Qiu, Xusheng Qiu, Lei Tan, Cuiping Song, Yingjie Sun, Ying Liao, Xiufan Liu, Chan Ding

**Affiliations:** a Shanghai Veterinary Research Institute, Chinese Academy of Agricultural Sciences, Shanghai, China; b School of Food and Bioengineering, Wuhu Institute of Technology, Wuhu, China; c School of Veterinary Medicine, Yangzhou University, Yangzhou, China; d Jiangsu Co-innovation Center for Prevention and Control of Important Animal Infectious Diseases and Zoonoses, College of Veterinary Medicine, Yangzhou University, Yangzhou, China; Changchun Veterinary Research Institute

**Keywords:** Newcastle disease virus, single cell, chicken, interferon response, transcript

## Abstract

Newcastle disease virus (NDV) is an avian paramyxovirus that causes major economic losses to the poultry industry around the world, with NDV pathogenicity varying due to strain virulence differences. However, the impacts of intracellular viral replication and the heterogeneity of host responses among cell types are unknown. Here, we investigated the heterogeneity of lung tissue cells in response to NDV infection *in vivo* and that of the chicken embryo fibroblast cell line DF-1 in response to NDV infection *in vitro* using single-cell RNA sequencing. We characterized the NDV target cell types in the chicken lung at the single-cell transcriptome level and classified cells into five known and two unknown cell types. The five known cell types are the targets of NDV in the lungs with virus RNA detected. Different paths of infection in the putative trajectories of NDV infection were distinguished between *in vivo* and *in vitro*, or between virulent Herts/33 strain and nonvirulent LaSota strain. Gene expression patterns and the interferon (IFN) response in different putative trajectories were demonstrated. IFN responses were elevated *in vivo*, especially in myeloid and endothelial cells. We distinguished the virus-infected and non-infected cells, and the Toll-like receptor signaling pathway was the main pathway after virus infection. Cell-cell communication analysis revealed the potential cell surface receptor-ligand of NDV. Our data provide a rich resource for understanding NDV pathogenesis and open the way to interventions specifically targeting infected cells.

**IMPORTANCE** Newcastle disease virus (NDV) is an avian paramyxovirus that causes major economic losses to the poultry industry around the world, with NDV pathogenicity varying due to strain virulence differences. However, the impacts of intracellular viral replication and the heterogeneity of host responses among cell types are unknown. Here, we investigated the heterogeneity of lung tissue cells in response to NDV infection *in vivo* and that of the chicken embryo fibroblast cell line DF-1 in response to NDV infection *in vitro* using single-cell RNA sequencing. Our results open the way to interventions specifically targeting infected cells, suggest principles of virus-host interactions applicable to NDV and other similar pathogens, and highlight the potential for simultaneous single-cell measurements of both host and viral transcriptomes for delineating a comprehensive map of infection *in vitro* and *in vivo*. Therefore, this study can be a useful resource for the further investigation and understanding of NDV.

## INTRODUCTION

Newcastle disease (ND) is a major disease that causes economic losses to the poultry industry (1, 2). ND was first documented in Newcastle-upon-Tyne, England, and Java, Indonesia, in the mid-1920s (3, 4). Within a few years, ND spread throughout the world and became endemic in many countries (5). Newcastle disease virus (NDV) belongs to the *Avulavirinae*, a subfamily of the *Paramyxoviridae* (6). The virus has an RNA genome with the structural proteins fusion protein (F), phosphoprotein (P), and hemagglutinin-neuraminidase (HA); a large polymerase protein (L); a matrix protein (M); and a nucleoprotein (NP) (7). More than 240 avian species can be infected by NDV by natural or experimental routes (8). NDV strains can be classified as highly virulent (velogenic), intermediately virulent (mesogenic), or nonvirulent (lentogenic) according to the intracerebral pathogenicity index (ICPI) in 1-day-old specific-pathogen-free (SPF) chickens (1). Lentogenic and mesogenic NDV strains are often used for the development of anti-NDV vaccines.

The surface of the mucous membrane of the chicken respiratory tract is one of the main targets for infection and penetration by respiratory pathogens such as NDV. The site of infection (eyes/nose) is close to the lungs, home to the bronchus-associated lymph tissue. The innate and adaptive immune responses of the respiratory tissues, including those of the lungs, are essential to prevent the virus from spreading to the rest of the body (9).

An understanding of the interactions between NDV and its host cells is critical for the development of successful preventive and therapeutic approaches. In several studies, the host response to NDV infection has been characterized by measuring bulk cell populations and their further experimental validation (10–13). However, *in situ* infection is far more complex than these rough models of host responses. For example, the specific cell type in the lungs targeted by NDV and the heterogeneity of the host responses are still unknown. Only a subset of cells had the virus, and most cells in the population were exposed but not infected and usually responded to defense signals such as type I interferons (IFNs).

Several key questions related to NDV infection have yet to be answered. In particular, the extent and nature of intracellular infection in different cell types have not been systematically elucidated. In addition, the heterogeneity of the host responses in bystander (uninfected) and infected cells across various cell types has not been systematically characterized. High-throughput single-cell RNA sequencing (scRNA-seq) has enabled the study of viral infection at unprecedented resolution (14–22). By quantifying viral RNA within cells, scRNA-seq enables the comparison of gene expression between infected and uninfected (bystander) cells in an infected host, providing a more accurate view of host and viral gene expression within infected cells. This method can also separate the direct influence of intracellular infection from the influence of the IFN response and quantify the cell type composition and expression programs of individual cell signals, which are obscured in bulk measurements.

Here, we provide a comprehensive single-cell atlas of NDV in chickens both *in vitro* and *in vivo*. We used a microfluidic scRNA-seq platform (10× Genomics) to analyze eight samples from chickens with NDV challenge both *in vitro* and *in vivo*, including six lung samples *in vivo* from SPF chickens infected with the highly virulent NDV Herts/33 strain or the nonvirulent LaSota strain and two DF-1 cell line samples infected with the highly virulent NDV Herts/33 strain *in vitro*. The cell types in the chicken lung targeted by NDV were characterized, and the putative trajectories of NDV infection were constructed to model gene expression changes and IFN responses with viral infection. Moreover, since NDV has an RNA genome and transcribes polyadenylated mRNAs, we detected viral RNA in individual cells and analyzed the potential cell surface receptor-ligand interactions. This enabled us to define NDV tropism at high resolution and identify NDV-related transcriptional changes in the antiviral response. These data help us to study host-virus interactions and comprehensively catalog changes in cell type abundance and cell states after NDV infection.

## RESULTS

### Integrated analysis of NDV scRNA-seq data.

**FIG 1:**
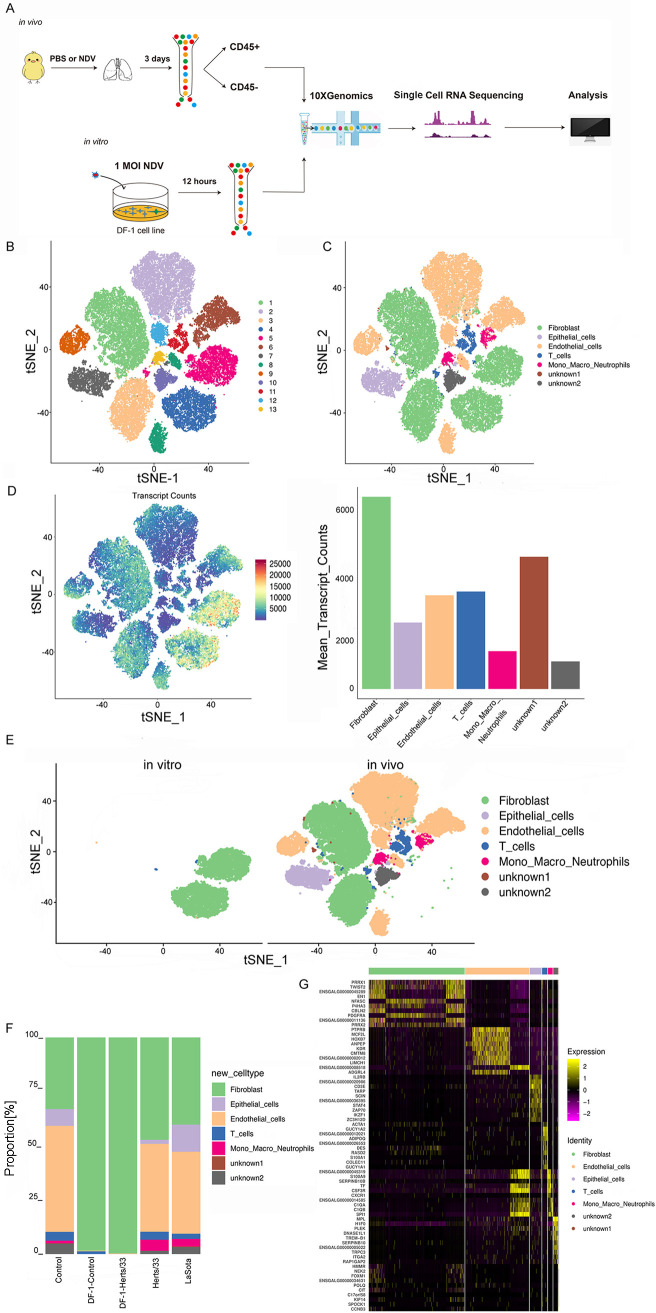
Global single-cell transcriptional landscape in the chicken lung and tbe DF-1 cell line by scRNA-seq and cell cluster identification. (A) Study design. Single cells from the lungs and the DF-1 cell line were sorted and subjected to scRNA-seq (droplet based; 10× Genomics). CD45 is used to distinguish the immune cells and non-immune cells. (B) t-SNE visualization of cells from the lungs and the DF-1 cell line, with 13 distinct clusters colored. (C) t-SNE visualization of the associated cell type detected in that cell. (D) t-SNE visualization of cells profiled with the number of transcripts (UMIs) detected in that cell and the number of cells and expressed transcript counts detected in each cell type. (E) t-SNE visualization of cells both *in vitro* and *in vivo*. (F) Bar plots showing the proportion of each cluster both *in vitro* and *in vivo* in the control, Herts/33, LaSota, DF-1–control, and DF-1–Herts/33 groups. (G) Heatmap of the expression of the top 10 DEGs in each cell type.

To generate a comprehensive single-cell atlas of NDV both *in vitro* and *in vivo*, we isolated six lung samples from SPF chickens infected with the highly virulent NDV Herts/33 strain or the nonvirulent LaSota strain for 3 days and DF-1 cell line samples infected with the highly virulent NDV Herts/33 strain for 12 h at a multiplicity of infection (MOI) of 1. Magnetically activated cell sorting (MACS) was used to isolate immune (CD45^+^) and nonimmune (CD45^−^) cells derived from the lungs of NDV-treated and phosphate-buffered saline (PBS) (control)-treated SPF chickens. Next, single-cell suspensions from the lungs and DF-1 cell lines were submitted to the 10× Genomics platform for scRNA-seq, which profiles all polyadenylated mRNAs (Fig. 1A). After stringent quality filtering, ~4 billion unique transcripts were obtained from 91,453 cells, in which over 1,300 genes and 50,284 mean reads per cell on average could be detected as being expressed (see Table S1 in the supplemental material). Subsequent dimensionality reduction and cluster analysis identified 13 clusters, which could be assigned to T, monocyte-macrophage-neutrophil (mono-macro-neutrophil), epithelial, fibroblast, and endothelial cells according to the specific markers selected, and red blood cells and doublets were excluded from the *t*-distributed stochastic neighbor embedding (t-SNE) map (Fig. 1B and C and Table S1). Moreover, these 13 clusters were composed of different cell types (Fig. S1A and J) and were from different samples (Fig. S1D and F to I). A list of marker genes used to identify each cell phenotype is provided in Table S1. Cell numbers, proportions, and detected transcript counts for each cell type are presented in Fig. 1D and F. Cluster bias was observed in cell samples *in vivo* and *in vitro* (Fig. 1E and Fig. S1E). For the distribution of cell types in each sample, the cell composition of each cell type after NDV infection was changed both *in vitro* and *in vivo* (Fig. S1B). *In vivo*, the proportion of fibroblast and mono-macro-neutrophil cells increased, especially the proportion of mono-macro-neutrophil cells remarkably increased after Herts/33 and LaSota infection. The proportion of epithelial, endothelial, and T cells decreased after Herts/33 infection. The proportion of epithelial cells increased and that of endothelial and T cells decreased after LaSota infection. *In vitro*, the proportion of fibroblast cells increased remarkably after Herts/33 infection. The composition of each sample for each cell type is shown in Fig. S1C. The details of the proportions of each cell type in each sample are shown in Fig. S1D, H, and I. A heatmap analysis of the top 10 markers showed the global transcriptional landscape and distinct signatures (Fig. 1G).

We describe the heterogeneity that underlies each cell type in more detail below.

### Phenotypic heterogeneity of fibroblast cells.

**FIG 2:**
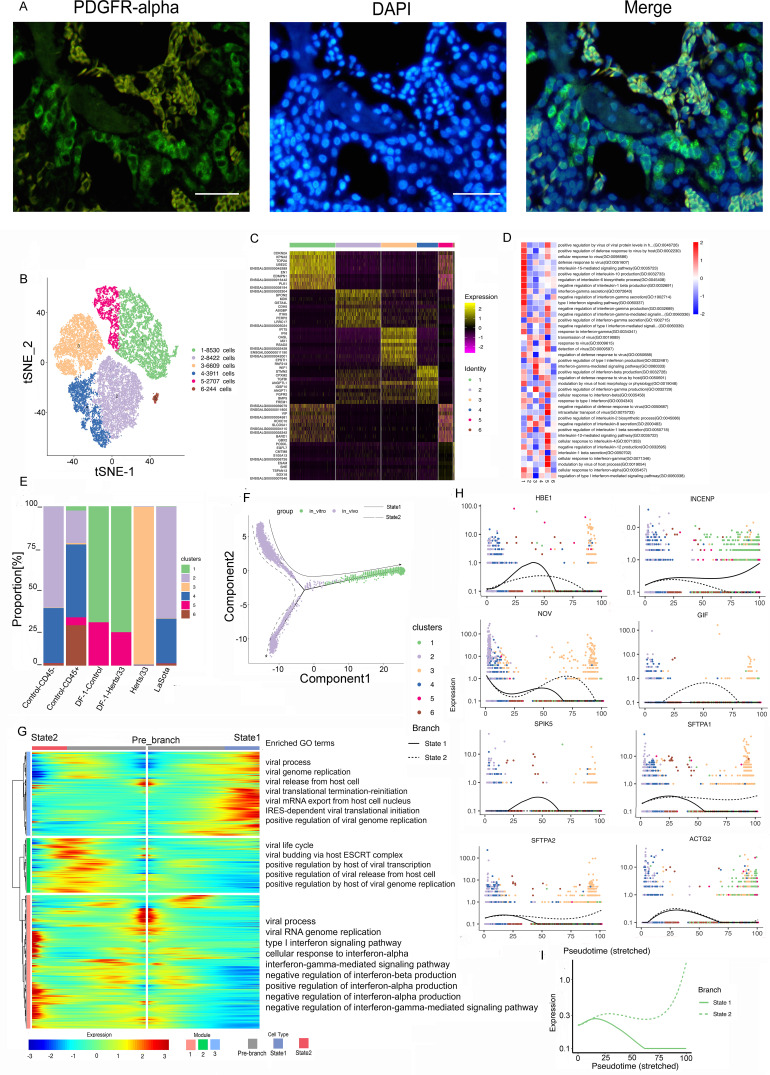
Fibroblast cell clusters in the lungs and the DF-1 cell line. (A) Immunostaining of PDGFR in the lungs. Scale bars, 20 μm. (B) t-SNE plot of 30,423 fibroblast cells color-coded by their associated clusters. (C) Heatmap of the expression of the top 10 DEGs in each cell cluster. (D) Differences in pathway activities scored per cell by GSVA between the different fibroblast clusters, with enriched GO terms (*P < *0.05). (E) Bar plots showing the proportion of each cluster both *in vitro* and *in vivo* in the control, Herts/33, LaSota, DF-1–control, and DF-1–Herts/33 groups. (F) Pseudotime trajectory plot representing NDV infection features both *in vitro* and *in vivo*. The solid line indicates features *in vitro*, and the dotted line indicates features *in vivo*. (G) Gene expression dynamics model along *in vitro* and *in vivo* lineages. (H) Expression patterns of the top 10 most dynamic genes in two states over pseudotime. (I) Dynamics of the IFN responses in two states over pseudotime.

Fibroblasts were the most frequent cell type. Immunofluorescence assays for platelet-derived growth factor receptor (PDGFR) further confirmed the presence of fibroblast cells in the lungs (Fig. 2A). We retrieved 30,423 fibroblast cells, which were separated into six clusters (Fig. 2B). These six identified clusters were from different samples (Fig. S2A, E, and F). In addition, the different samples were composed of different clusters (Fig. 2E and Fig. S2C, G, and H). The cell composition of each cluster after NDV infection was changed both *in vivo* and *in vitro* (Fig. 2E). Cluster bias was observed in cell samples *in vivo* and *in vitro* (Fig. S2D).

*In vivo*, clusters 2 to 6 were the majority in the control sample, cluster 3 comprised the main proportion in the Herts/33 sample, and clusters 2 and 4 comprised the main proportions in the LaSota sample. *In vitro*, clusters 1 and 5 were mainly in the DF-1 cell line sample. The proportion of cluster 5 increased after Herts/33 infection compared with the control. A heatmap analysis of the top 10 markers showed that clusters 1 and 5 had similar expression patterns (Fig. 2C). Thus, clusters 1 and 5 could have similar functions and could be attributed to the same cell subtype in fibroblasts. Furthermore, the function of every cluster was analyzed using gene set enrichment analysis (GSEA). The six clusters were enriched mainly in different Gene Ontology (GO) terms and KEGG pathways focusing on virus, IFN, or inflammatory responses (Fig. 2D and Fig. S2I).

To understand the changes in the cluster state after NDV infection, we used Monocle to infer pseudotime trajectories. We observed three distinct trajectories. A different pseudotemporal infection trajectory was observed in the putative state transition directions. Two major bifurcations were observed in the trajectory analysis (Fig. 2F). Combined with the pseudotime analysis, we could conclude the states and relationships among the pseudotemporal infection trajectories of fibroblast cells both *in vitro* and *in vivo*. Branch state 3 to branch state 1 was the putative trajectory of NDV infection *in vitro*, and branch state 3 to branch state 2 was the putative trajectory of NDV infection *in vivo* (Fig. S2K to N). State 3 was the beginning of the trajectory and was composed mainly of clusters 2, 3, and 4 (Fig. S2J). State 2 was comprised mainly of clusters 3, 4, and 6. State 1 consisted mainly of clusters 1 and 5 (Fig. S2J). These results were consistent with the cluster composition in each sample both *in vitro* and *in vivo*.

To further unravel the different regulating modules of gene expression during the infection trajectories both *in vitro* and *in vivo*, we next attempted to identify key molecular changes in a pseudotime-dependent manner that play important roles along two major bifurcations in fibroblast cells. We then modeled gene expression along two major bifurcations and identified three gene expression modules with specific expression (Fig. 2G). To help in determining the biological processes of fibroblast cells in viral infection, GO term enrichment analysis was performed for three gene expression modules (Fig. 2G). Module 1 comprised mainly gene sets that were highly enriched from the prebranch state to state 2. Among these gene sets, the majority were involved in the biological process of IFN response and regulation (e.g., STAT1, ADAR, IFIH1, and IFITM1). Genes enriched in module 2 were highly expressed only in state 2 and were involved in the positive regulation of viral transcription in the host, viral genome replication in the host, and viral release from host cells, i.e., the viral life cycle (e.g., CHMP4B, VPS37B, VPS4B, and UBC). Module 3 contained genes that were highly expressed in state 1, with most genes being enriched in the viral process, including viral genome replication, virus release from host cells, viral mRNA (vmRNA) export from host cells, internal ribosome entry site (IRES)-dependent viral translational initiation, the positive regulation of viral genome replication, and viral translational termination-reinitiation (e.g., EIF3L, TARBP2, KARS, and EIF3B).

Distinct gene regulation patterns were constructed to reflect the viral infection state after NDV infection both *in vitro* and *in vivo* in fibroblast cells. Modules 1 and 2 represent the viral infection state *in vivo*, and module 3 represents the viral infection state *in vitro*. Next, we identified the top 50 greatly variable genes in the putative trajectory of fibroblast cells, including SFTPA1, SFTPA2, SPIK5, ACTG2, LYG2, IFIH1, IFITM1, CSRP2, MYLK, and IGLL1 in module 1; NOV, S100A9, and NOS2 in module 2; and INCENP, TAGLN, and virus (GenBank accession number AY741404.1) expression in module 3. These genes showed different expression patterns under different states over pseudotime (Fig. 2H). These results suggests that *in vivo* and *in vitro* gene expression differed over pseudotime. To further investigate the IFN response after NDV infection, the IFN response was analyzed in the putative trajectory of fibroblast cells. The IFN response showed a drastic difference, with a significant increase in state 2 and an obvious reduction in state 1 (Fig. 2I). These results were consistent with the results of GO enrichment in the module analysis. Thus, these results suggest that the IFN responses in fibroblast cells were different *in vitro* and *in vivo*. The IFN response gradually intensified *in vivo*, but it declined *in vitro*.

### Phenotypic heterogeneity of myeloid cells.

**FIG 3:**
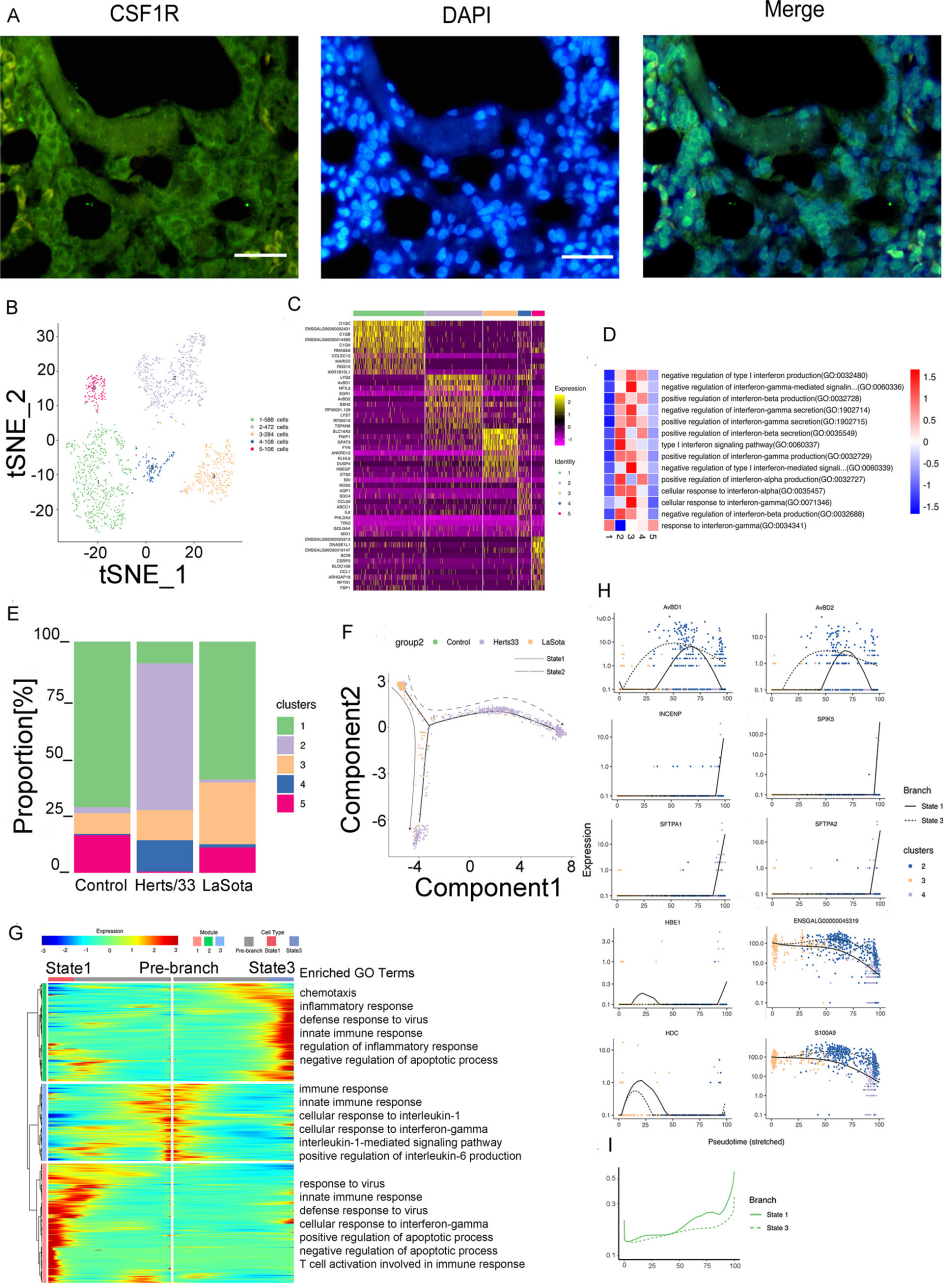
Myeloid cell clusters in the lungs. (A) Immunostaining of CSF1R in the lungs. Scale bars, 20 μm. (B) t-SNE plot of 1,588 mono-macro-neutrophil cells color-coded by their associated clusters. (C) Heatmap of the expression of the top 10 DEGs in each cell cluster. (D) Differences in pathway activities scored per cell by GSVA between myeloid cells isolated from the lungs, with enriched GO terms (*P < *0.05). (E) Bar plots showing the proportion of each cluster *in vivo* in the control, Herts/33, and LaSota groups. (F) Pseudotime trajectory plot representing NDV infection features of the highly virulent NDV Herts/33 strain or the nonvirulent LaSota strain. The solid line indicates features of the nonvirulent LaSota strain, and the dotted line indicates features of the highly virulent Herts/33 strain. (G) Gene expression dynamics model for the nonvirulent LaSota strain and the highly virulent NDV Herts/33 strain. (H) Expression patterns of the top 10 most dynamic genes in two states over pseudotime. (I) Dynamics of the IFN responses in two states over pseudotime.

Myeloid cells were detected *in vivo*. Immunofluorescence assays for CSF1R further confirmed the presence of myeloid cells in the lungs (Fig. 3A). Five clusters were identified in 1,558 myeloid cells *in vivo* (Fig. 3B and Fig. S3B). These five identified clusters were obtained from different samples (Fig. S3A). In addition, the different samples were composed of different clusters (Fig. 3E and Fig. S3C, E, and F). The cell composition of each cluster after NDV infection was changed. The proportions of clusters 2 to 4 significantly increased and those of clusters 1 and 5 significantly decreased after Herts/33 infection compared with the control. In the LaSota sample, the proportion of cluster 3 significantly increased and that of cluster 4 increased slightly, but the proportions of clusters 1, 2, and 5 decreased compared with the control (Fig. 3E).

The top 10 marker genes with distinct signatures in each cluster are shown in Fig. 3C. Furthermore, we analyzed the function of every cluster using GSEA. The five clusters were enriched mainly in different GO terms and KEGG pathways focusing on the virus, IFN, or inflammatory response (Fig. 3D and Fig. S3G).

Because myeloid cells usually include neutrophils, monocytes, and macrophages, we attempted to use human or mouse cell markers of myeloid cells to identify each cluster and distinguish the cell subtypes in myeloid cells. The marker gene sets were used to identify each cluster, combined with manual correction using Loupe Browser6 and integrating the gene set variation analysis (GSVA) results. Clusters 1 and 5 were excluded from the subsequent pseudotime analysis because they might have been neutrophils (Fig. S3H). Overall, three distinct trajectories were observed. We observed a different pseudotemporal infection trajectory in the putative state transition directions. Two major bifurcations were observed in the trajectory analysis (Fig. 3F). Combined with the results of the pseudotime analysis, we could infer the states and relationships among the pseudotemporal infection trajectories in monocyte-macrophage cells. State 1 contained mainly LaSota-infected cells. State 3 was composed mostly of Herts/33-infected cells. State 2 was the beginning of the trajectory and comprised control and Herts/33-infected cells. Therefore, branch state 2 to branch state 1 was the putative trajectory of LaSota infection, and branch state 2 to branch state 3 was the putative trajectory of Herts/33 infection (Fig. S3I to K).

Three gene expression modules were identified along these two trajectories in the differential gene expression analysis (Fig. 3G). Module 1 mainly comprised gene sets that were highly enriched only in state 1. Among these gene sets, most genes were involved in the biological processes of response to virus (e.g., OASL, ZC3HAV1, and IFIH1), defense response to virus (e.g., IRF1, OASL, MX1, and SAMHD1), regulation of apoptosis (e.g., MERTK, IL6, RPS29, EGFR, and PLK2), and the innate immune response (e.g., SAMHD1, IFIH1, OASL, and ZC3HAV1). The genes enriched in module 2 were highly expressed in state 3 and were involved in the defense response to virus (e.g., STAT1, IRF7, and RSAD2), the innate immune response (e.g., NLRC5, RSAD2, HEXIM1a, and IFIT5), and the inflammatory response (e.g., SEMA7A, IL8, PTGS2, and RICTOR). Module 3, containing genes that were highly expressed in the prebranch state, was involved in the innate immune response (e.g., TLR4 [Toll-like receptor 4], TLR15, S100A9, and HMGB1) and the cellular response to interleukin-1 (IL-1) and the IL-1-mediated signaling pathway (e.g., FN1, IL1RAP, RC3H1, and IRAK2). In summary, the common and distinct gene regulation patterns were constructed for monocyte-macrophage differentiation in NDV infection, which contributed to an in-depth understanding of NDV infection processes and the underlying regulatory mechanism. These results demonstrated the cell response to highly virulent NDV Herts/33 strain or nonvirulent LaSota strain infection in myeloid cells.

Next, we identified the top 50 greatly variable genes in the putative trajectory of monocyte-macrophage cells, including SFTPA1, SFTPA2, INCENP, SPIK5, and HBE1 in module 1; AvBD1 and AvBD2 in module 2; and ENSGALG00000045319, HDC, and S100A9 in module 3. These genes showed different expression patterns under different states over pseudotime (Fig. 3H). To further investigate the antiviral response after NDV infection, the IFN response was analyzed in the putative trajectory of monocyte-macrophage cells. The IFN response was significantly increased in both states 1 and 3 (Fig. 3I). This result was consistent with the results of GO enrichment in the module analysis. Therefore, either highly virulent NDV Herts/33 strain infection or nonvirulent LaSota strain infection can cause a significant IFN response in monocyte-macrophage cells.

### Phenotypic heterogeneity of endothelial cells.

**FIG 4:**
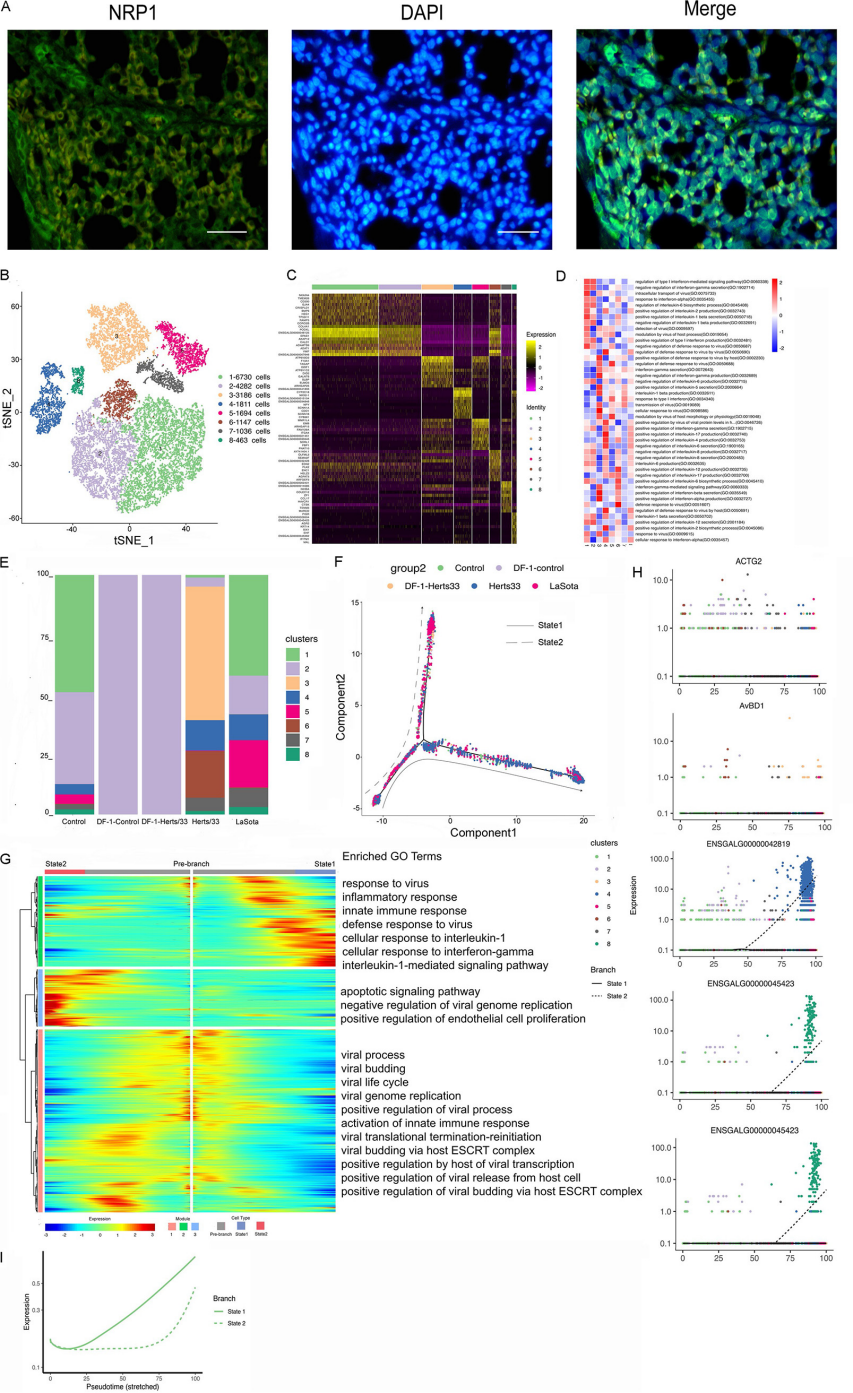
Endothelial cell clusters in the lungs and the DF-1 cell line. (A) Immunostaining of NRP1 in the lungs. Scale bars, 20 μm. (B) t-SNE plot of 20,349 endothelial cells color-coded by their associated clusters. (C) Heatmap of the expression of the top 10 DEGs in each cell cluster. (D) Differences in pathway activities scored per cell by GSVA between endothelial cells isolated from different epithelial clusters, with enriched GO terms (*P < *0.05). (E) Bar plots showing the proportion of each cluster both *in vitro* and *in vivo* in the control, Herts/33, LaSota, DF-1–control, and DF-1–Herts/33 groups. (F) Pseudotime trajectory plot representing NDV infection features of the highly virulent NDV Herts/33 strain or the nonvirulent LaSota strain. The solid line indicates features of the highly virulent Herts/33 strain, and the dotted line indicates features of the nonvirulent LaSota strain. (G) Gene expression dynamics model for the highly virulent NDV Herts/33 strain and the nonvirulent LaSota strain. (H) Expression patterns of the top 10 most dynamic genes in two states over pseudotime. (I) Dynamics of the IFN responses in two states over pseudotime.

Immunofluorescence assays for NRP1 confirmed the presence of endothelial cells in the lungs (Fig. 4A). A total of 20,349 endothelial cells were detected, including 20,320 cells *in vivo* and 29 cells *in vitro*, and reclustering revealed eight clusters (). These eight identified clusters were from different samples (Fig. S4A, E, and F). In addition, different samples were composed of different clusters (Fig. 4E and Fig. S4C, E, and F). Cluster bias was observed in cell samples *in vivo* and *in vitro* (Fig. 4E and Fig. S4D)*. In vitro*, the endothelial cells were composed of only cluster 2. *In vivo*, clusters 1, 2, 4, 5, 7, and 8 were composed of the cell subtype in the control group. After Herts/33 infection, the proportions of clusters 1, 2, and 5 were significantly decreased, and those of clusters 4, 6, and 7 were increased, with cluster 3 appearing. In the LaSota sample, the proportions of clusters 1 and 2 were decreased, and those of clusters 4, 5, 6, 7, and 8 were increased compared with the control. *In vitro*, there was no change in the cell composition after NDV infection. The composition details for each cell cluster in each sample, including CD45^+^ or CD45^−^ cells, are shown in Fig. S4H and I.

Based on a heatmap analysis of the top 10 markers, clusters 1, 2, and 6 exhibited similar expression patterns (Fig. 4C). Thus, clusters 1, 2, and 6 could have similar functions and could be attributed to the same cell subtype in endothelial cells. The function of each cluster was then investigated using GSEA. Viral response, IFN response, and inflammatory response were the most abundant GO terms and KEGG pathways in the eight clusters (Fig. 4D and Fig. S4J).

Thereafter, we used Monocle to infer pseudotime trajectories and examine how the cluster state evolved after NDV infection. Three distinct trajectories were observed. In the putative state transition directions, we found a distinct pseudotemporal infection path. A trajectory study revealed two main bifurcations (Fig. 4F). Cell subtypes were not changed, and there were few cells after NDV infection *in vitro*. This putative trajectory presents the possible trajectory after NDV infection *in vivo*. Most LaSota-infected cells were in state 2, which had few control cells. State 3 contained mainly control cells. Most Herts/33-infected cells were in state 1, which had few control cells (Fig. S4L to O). Therefore, branch state 3 to branch state 1 could be the putative trajectory of Herts/33 infection, and branch state 3 to branch state 2 could be the putative trajectory of LaSota infection. Three gene modules were recognized along these putative trajectories in the differential gene expression analysis (Fig. 4G). Module 1, containing genes that were upregulated in the prebranch state and in the direction to state 2, was involved in viral processes, including the viral process (e.g., MAVS, RICTOR, NUP155, and RCOR1), the viral life cycle (e.g., UBC, MVB12B, VPS37B, and TSG101), viral budding (e.g., TSG101, LRSAM1, and CHMP5), and viral translational termination-reinitiation (e.g., EIF3L, EIF3D, EIF3G, and EIF3B). Module 2, containing genes that were upregulated in state 1, was involved in the defense response to virus (e.g., STAT1, TBK1, IL6, OASL, RSAD2, ADAR, IRF7, and IFIH1), the innate immune response (e.g., DDX21, TBK1, OASL, RSAD2, ADAR, and IFIH1), and the inflammatory response (e.g., IL10, THEMIS2, IL18, and IL15). Module 3, containing genes that were upregulated in state 2, was involved in the negative regulation of viral genome replication (e.g., IFITM1, SRPK2, SPIK5, and RNASEL), the positive regulation of endothelial cell proliferation (e.g., CDH13, VEGFA, PDCL3, PGF, and SEMA5A), and the apoptotic signaling pathway (e.g., PAWR, DAP, DAP3, and NDUFA1). In summary, distinct gene regulation patterns were constructed to reflect the viral infection state after NDV infection.

We also examined the top 50 greatly variable genes in endothelial cells along the putative trajectory, including HBE1, MYH11, ENSGALG00000032344, HBA1, and CSRP2 in module 1; AvBD1, AvBD2, ACTG2, CCL1, SPP1, and virus (GenBank accession number AY741404.1) expression in module 2; and SFTPA1, SFTPA2, INCENP, and SPIK5 in module 3. Various expression patterns were observed between states over pseudotime (Fig. 4H). This result suggests that gene expression differs in the highly virulent NDV Herts/33 strain or the nonvirulent LaSota strain over pseudotime. To further investigate the antiviral response after NDV infection, the IFN response was analyzed in the putative trajectory of endothelial cells. The IFN response was significantly increased in state 1, and it increased in state 2 at a later stage (Fig. 4I). Thus, infection with either the highly virulent NDV Herts/33 strain or the nonvirulent LaSota strain can cause a significant antiviral response in endothelial cells, although they present different IFN response patterns.

### Phenotypic heterogeneity of epithelial cells.

**FIG 5:**
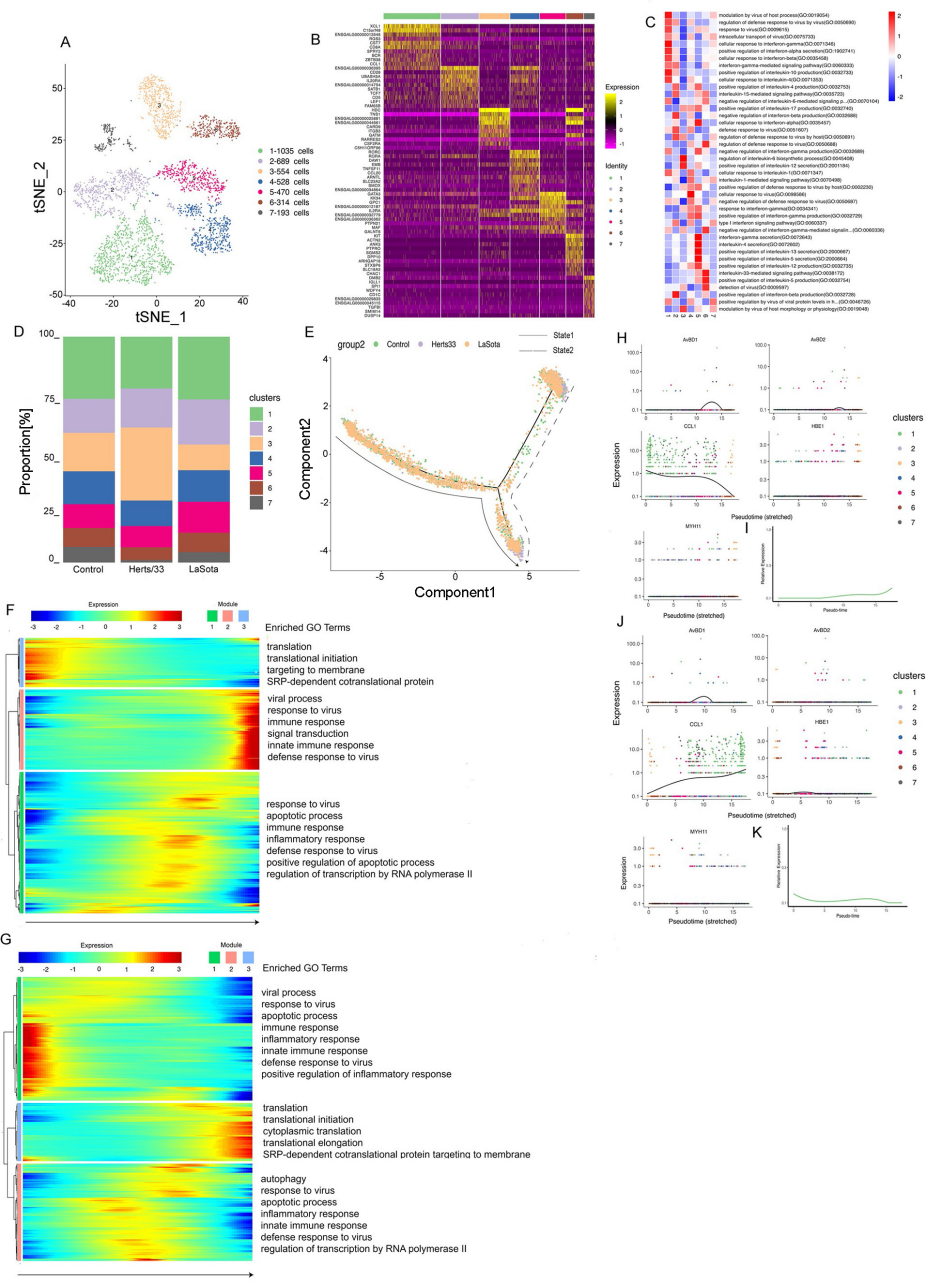
Epithelial cell clusters in the lungs. (A) t-SNE plot of 3,783 epithelial cells color-coded by their associated clusters. (B) Heatmap of the expression of the top 10 DEGs in each cell cluster. (C) Differences in pathway activities scored per cell by GSVA between epithelial cells isolated from different epithelial clusters, with enriched GO terms (*P < *0.05). (D) Bar plots showing the proportion of each cluster both *in vitro* and *in vivo* in the control, Herts/33, and LaSota groups. (E) Pseudotime trajectory plot representing NDV infection features of the highly virulent NDV Herts/33 strain or the nonvirulent LaSota strain. The solid line indicates features of the highly virulent Herts/33 strain, and the dotted line indicates features of the nonvirulent LaSota strain. (F and G) Gene expression dynamics model for the highly virulent NDV Herts/33 strain and the nonvirulent LaSota strain. (H and J) Expression patterns of the top 10 most dynamic genes in two states over pseudotime. (I and K) Dynamics of the IFN responses in two states over pseudotime.

We retrieved 3,783 epithelial cells *in vivo*, which were divided into seven clusters (Fig. 5A and Fig. S5B). These seven identified clusters were obtained from different samples (Fig. S5A). Next, we assessed the prevalence of each epithelial cluster in each sample. All seven clusters were in the control group. After Herts/33 infection, the proportion of cluster 3 remarkably increased, and those of clusters 4, 6, and 7 decreased compared with the control. After LaSota infection, the proportion of cluster 3 significantly decreased, and those of clusters 2 and 5 increased compared with the control. Therefore, epithelial cells showed different cell cluster patterns after infection with the highly virulent NDV Herts/33 strain or the nonvirulent LaSota strain. Figure 5D and Fig. S5C to F show the precise composition of each cell cluster in each sample.

According to a heatmap analysis of the top 10 markers, clusters 2 and 4 exhibited similar expression patterns (Fig. 5B). This finding showed that clusters 2 and 4 could have similar functions and could belong to the same cell subtype. Therefore, we next investigated the function of each cluster using GSEA. The most common GO terms and KEGG pathways in the seven clusters were viral response, IFN response, and inflammatory response (Fig. 5C and Fig. S5G).

Using pseudotime analysis, three states were observed under varied viral infections (Fig. S5I to K). State 1 included the control and LaSota groups. State 2 included the control, Herts/33, and LaSota groups. State 3 also included the control, Herts/33, and LaSota groups, but the proportion of the Herts/33 group was decreased. We inferred two alternative putative trajectories based on the proportions of Herts/33 cells increasing and LaSota cells decreasing under varied NDV infection. Thus, one direction was state 1 to state 2, and the other direction was state 3 to state 2 (Fig. 5E). Therefore, the two directions can be attributed to the putative state of Herts/33 or LaSota over pseudotime. Branch state 1 to branch state 2 included clusters 1, 2, 4, 5, and 7, and branch state 3 to branch state 2 included clusters 3 and 6 (Fig. S5H).

Three gene modules were found in the two putative trajectories in the differential gene expression analysis (Fig. 5F and G). In branch state 1 to branch state 2, module 1 was involved in the viral process (e.g., MAVS, RICTOR, NUP155, and RCOR1), the inflammatory response (e.g., IL8L1, IL17A, ACOD1, CCL17, and IL8), apoptosis (e.g., IL4I1, TNIP2, RHOB, FOXO1, HIP1R, and PIM1), and the immune response (e.g., IL8L1, NFIL3, IL8, IL1B, and DMB2). Module 2 genes were highly expressed in state 2 and were involved in the viral process (e.g., ZC3HAV1, RSAD2, and GATA3), the innate immune response (e.g., DDX21, TBK1, OASL, RSAD2, ADAR, and IFIH1), signal transduction (e.g., TRAF1, TNFRSF18, RGS1, PLCG1, and RASAL2), antigen processing, and presentation of peptide antigen via major histocompatibility complex (MHC) class I (e.g., BF1 and BF2). Module 3 genes were highly expressed in state 1 and were involved in translation and translational initiation (e.g., RPL19, RPS10, and RPL27) and signal recognition particle (SRP)-dependent cotranslational protein targeting to the membrane (e.g., PL23A, RPS14, and RPL6).

For branch state 3 to branch state 2, the genes enriched in module 1 were highly expressed in state 3 and were involved in the viral process (e.g., ANKRD17, SPEN, and IFIH1), the immune response and the innate immune response (e.g., PLSCR1, FTH1, TRAF3, and OASL), and the inflammatory response and apoptosis (e.g., TRAF1, TRAF3, IFI6, and IRF1). In module 2, the genes enriched were highly expressed in state 2 and were involved in the response to virus (e.g., ZC3HAV1, RSAD2, and GATA3), the defense response to virus (e.g., MX1, RSAD2, and PMAIP1), apoptosis (e.g., OGT, SLK, TIA1, ROCK1, and TNIP2), the innate immune response (e.g., MX1, RSAD2, and ZC3HAV1), the inflammatory response (e.g., LY86, TNIP2, IL17A, CCL17, and EXFABP), and autophagy (e.g., PLEKHM1, FOXO1, and PTPN22). The genes in module 3 were enriched in terms associated with translation (Fig. 5G). The gene expression patterns and enriched GO terms were similar in these two trajectories. Therefore, these two trajectories represent the state of viral infection in epithelial cells. We also examined the top 50 greatly variable genes in epithelial cells along the putative trajectory. In state 1 to state 2, HBE1, AvBD1, MYH11, AvBD2, CCL20, HBM, S100A9, HBA1, IL6, CST3, and NOS2 in module 1; HDC, IFIH1, IL9, TGM4, and SRGN in module 2; and CCL1, IGLL1, and XCL1 in module 3 showed most expression variation. In state 3 to state 2, HBE1, MYH11, HDC, HBM, S100A9, HBA1, IFIH1, IL9, and virus (GenBank accession number AY741404.1) in module 1; AvBD1, AvBD2, SERPINB10B, COL1A2, NOS2, JCHAIN, CCL17, and RSAD2 in module 2; and CCL1, CCL20, IGLL1, XCL1, TARP, and RUNX3 in module 3 showed the most changes in expression. These genes also showed different expression patterns under both conditions (Fig. 5H and J). To examine the antiviral response after NDV infection, the IFN response was analyzed in the putative trajectories of epithelial cells. The two putative trajectories did not show a significant response to IFNs (Fig. 5I and K). This result was consistent with the results of the gene module analysis for the few IFN-stimulated genes (ISGs) observed in both states. Thus, NDV infection did not trigger the IFN response in epithelial cells.

### Phenotypic heterogeneity of T cells.

**FIG 6:**
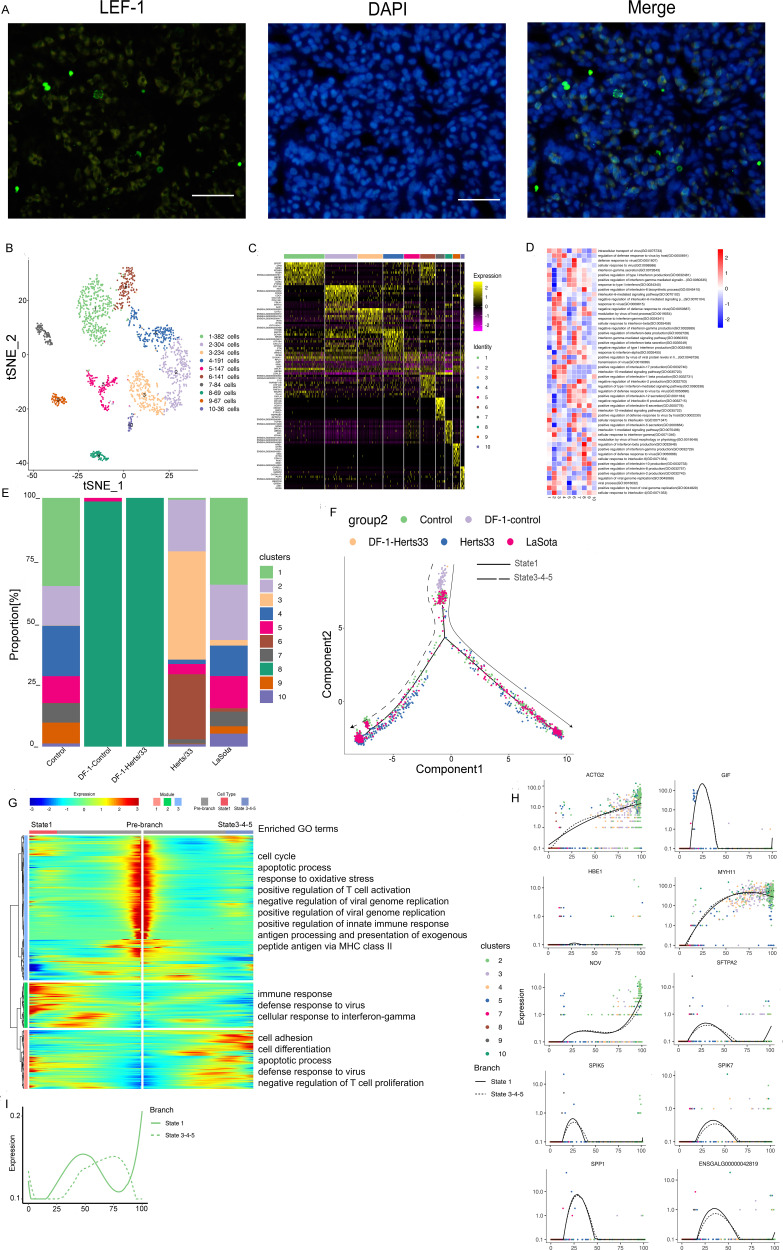
T cell clusters in the lungs and the DF-1 cell line. (A) Immunostaining of LEF1 in the lungs. Scale bars, 20 μm. (B) t-SNE plot of 1,655 epithelial cells color-coded by their associated clusters. (C) Heatmap of the expression of the top 10 DEGs in each cell cluster. (D) Differences in pathway activities scored per cell by GSVA between T cells isolated from different T cell clusters, with enriched GO terms (*P < *0.05). (E) Bar plots showing the proportion of each cluster both *in vitro* and *in vivo* in the control, Herts/33, LaSota, DF-1–control, and DF-1–Herts/33 groups. (F) Pseudotime trajectory plot representing NDV infection features of the highly virulent NDV Herts/33 strain or the nonvirulent LaSota strain. The solid line indicates features of the highly virulent Herts/33 strain, and the dotted line indicates features of the nonvirulent LaSota strain. (G) Gene expression dynamics model for the highly virulent NDV Herts/33 strain and the nonvirulent LaSota strain. (H) Expression patterns of the top 10 most dynamic genes in two states over pseudotime. (I) Dynamics of the IFN responses in two states over pseudotime.

Finally, we retrieved 1,655 T cells, including 1,585 cells *in vivo* and 70 cells *in vitro*, which were divided into 10 distinct clusters (Fig. 6B). Immunofluorescence assays for LEF1 further confirmed the presence of T cells in the lungs (Fig. 6A). These 10 clusters were obtained from different samples (Fig. S6A). The cluster details for each sample are shown in Fig. 6E and Fig. S6D to H. Clusters 5 and 8 were *in vitro* samples, and clusters 1 to 10 (except for cluster 8) were *in vivo* samples (Fig. S6B). *In vitro*, cluster 8 was in all Herts/33 samples, compared with the control, containing a majority of cluster 8 and a minority of cluster 5. *In vivo*, clusters 1, 2, 4, 5, 7, 9, and 10 were in the control sample; clusters 2, 3, 4, 5, 6, 7, 9, and 10 were in the Herts/33 sample; and clusters 1, 2, 3, 4, 5, 6, 7, 9, and 10 were in the LaSota sample. The proportions of each cluster were different in the Herts/33 and LaSota samples compared with the control sample. DEGs for each cluster were analyzed to decipher their unique gene expression signatures. A heatmap analysis of the top 10 markers showed the global transcriptional landscape and distinct signatures in each cluster (Fig. 6C). Thus, the 10 clusters could have distinct functions. We then used GSEA to examine the function of each cluster. The functions of the clusters focused on viral, IFN, and inflammatory responses. The most common GO terms and KEGG pathways are shown in Fig. 6D and Fig. S6I.

Furthermore, we examined the conditions between different viral infections using pseudotime analysis. Finally, five states were distributed on two branches (Fig. S6L). States 3 to 5 were combined into one state because they were on the same branch. The control, Herts/33, and LaSota samples were distributed in all states. Most Herts/33-infected cells were distributed mainly in state 3, and a few Herts/33-infected cells were distributed mainly in state 1. Most LaSota-infected cells were distributed mainly in state 1, and a few LaSota-infected cells were distributed mainly in state 3. State 2 was composed of control cells. For a few cells *in vitro*, the state did not change in the putative trajectories (Fig. S6M to O). Therefore, we speculated two directions. One direction was state 2 to state 1, and the other direction was state 2 to state 3. Branch state 2 to state 1 included clusters 1, 3, 4, 5, and 6, and branch state 2 to state 3 included clusters 2, 3, 4, and 10. The beginning of the branch included clusters 7, 8, and 9 (Fig. S6J). Therefore, the putative trajectory was the NDV infection state *in vivo* with two differentiation directions (Fig. 6F). One direction was dominated by the highly virulent Herts/33 strain, and the other was dominated by the nonvirulent LaSota strain. Next, three gene modules along two trajectories were identified in the differential gene expression analysis (Fig. 6G). Module 1, containing genes that were upregulated in state 3, was involved in the positive regulation of transcription by RNA polymerase II (e.g., ZNF821, IFT74, CREBRF, and PSMC3), the defense response to virus (e.g., IRF1, IFI6, OASL, IRF7, and MX1), cell differentiation (e.g., CTGF, DDX3X, and FGFR3), apoptosis (e.g., CDK11A, IRF1, BCAP29, and MINDY3), and the negative regulation of T cell proliferation (e.g., SDC4, PELI1, PAWR, and PDE5A). Module 2, containing genes that were upregulated in state 2, was involved in the immune response (e.g., CD36, IL8, IL1B, and CEBPG), the positive regulation of transcription by RNA polymerase II (e.g., ZNF593, DAB2IP, HELZ2, CD3D, INO80, and CYR61), the defense response to virus (e.g., CRCP, ZMYND11, STAT1, DTX3L, and RSAD2), and the cellular response to IFN-γ (e.g., FLNB, EVL, and ACOD1). Module 3, containing genes that were upregulated in the prebranch, was involved in the regulation of viral genome replication (e.g., IFITM1, SRPK2, ZC3HAV1, SPIK5, and LARP1), the positive regulation of the innate immune response (e.g., PLSCR1, HMGB3, and HMGB2), and the positive regulation of T cell activation (e.g., THY1, HSPD1, and FAM49B).

We then dissected the 50 main differentially communicated qualities in the putative trajectories of T cells, including ENSGALG00000042819, ACTG2, NOV, MYH11, SPIK7, ACTA1, IFIH1, S100A9, and virus (GenBank accession number AY741404.1) in module 1; HBE1, CCLI5, HDC, HBM, and HBA1 in module 2; and SFTPA2, SPIK5, GIF, SPP1, IFITM1, and ANKRD1 in module 3. Diverse expression patterns in the two diverse states were observed over pseudotime. To further explore the host response after NDV infection, the IFN response was investigated in the putative trajectories of T cells. The IFN response showed different patterns in the two states (Fig. 6H). Although the IFN response increased in both states at the earlier stage, the IFN response increased in state 1 and decreased in state 3 (Fig. 6I). State 1 included a majority of LaSota-infected cells, and state 3 included a majority of Herts/33-infected cells. Therefore, these results suggested that the IFN response was much stronger after LaSota infection than after Herts/33 infection. Thus, the nonvirulent LaSota strain can be used for vaccinating chickens.

### NDV RNAs detected in cell types.

**FIG 7:**
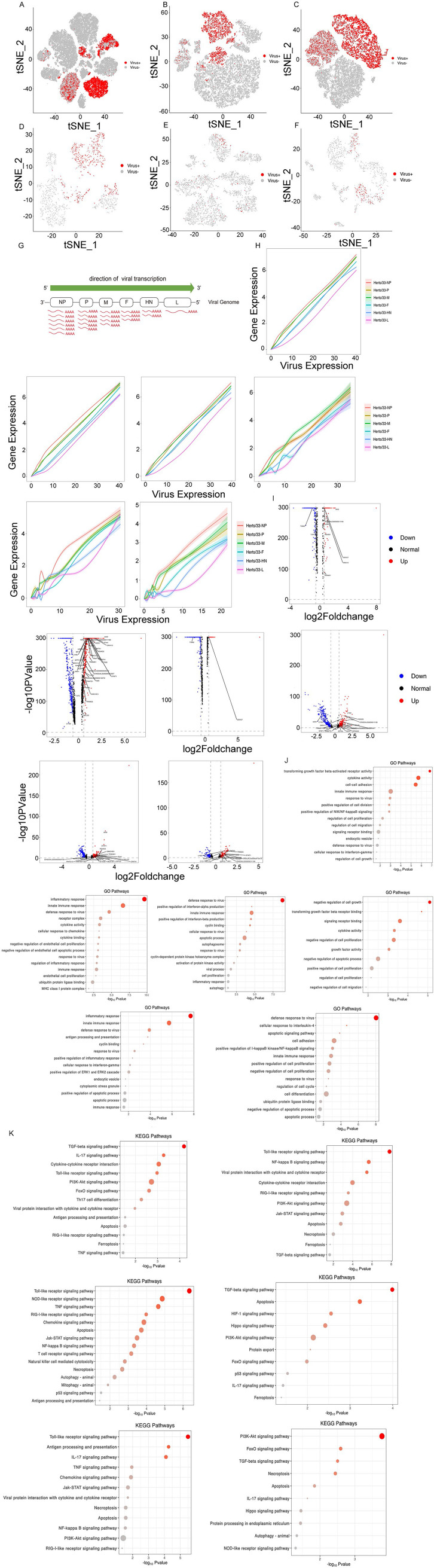
Cell types with highly virulent NDV Herts/33 strain RNA detected. (A) A total of 56,995 cells with Herts/33 RNA detected (UMI counts of >0) from the total cells both *in vitro* and *in vivo*. (B to F) Cells with Herts/33 RNA detected (UMI counts of >0) from fibroblast, myeloid, endothelial, epithelial, and T cells. (G) Schematic of NDV transcription. The viral RNA-directed RNA polymerase transcribes each gene sequentially but occasionally releases the genomic RNA template, ending transcription. Thus, the transcription frequency decreases from NP to L. (H) Proportion of each NDV gene versus the viral load from total, fibroblast, myeloid, endothelial, epithelial, and T cells. The *x* axis indicates the sum of the expression of six viral genes in each cell, and the *y* axis indicates the expression of each viral gene in each cell. (I) Volcano plots showing DEGs of virus-positive versus virus-negative cells from total, fibroblast, myeloid, endothelial, epithelial, and T cells. ISGs are shown in the plot (*P < *0.05). Red indicate up regulation and blue indicated down regulation. (J and K) GO and KEGG pathway analyses of IFN signaling and the response to virus signaling, comparing virus-positive to virus-negative cells from total, fibroblast, myeloid, endothelial, epithelial, and T cells.

Since vmRNA was polyadenylated, the 10× Genomics 3′ single-cell instrument captured both viral and host mRNAs within each cell. We discovered that the virulent Herts/33 strain was prevalent in infected cells within each of the five major cell types, but only the expression of M genes of the nonvirulent LaSota strain was detected in two fibroblast cells (Fig. 7A to C and Fig. S7A to E). The distribution and expression of each virus structural gene fragment in each major cell type are shown in Fig. 7D to F. Because few cells were detected with the fragment of the nonvirulent LaSota strain, the subsequent analysis was based on vmRNA in virulent Herts/33 strain infection.

We analyzed the distribution of NDV in each cell type. The virus infected epithelial, fibroblast, and T cells both *in vitro* and *in vivo*. *In vitro*, all DF-1 cells were infected with the virus. This suggests that the infection dose of the virus was an MOI of 1 at least (Fig. S7F). *In vivo*, the virus infection patterns in each cell type were different. The virus infected both CD45^+^ and CD45^−^ cells, but the proportion of CD45^+^ virus-positive cells was higher than that of CD45^−^ virus-positive cells (Fig. S7G to I). The virus infected mono-macro-neutrophil and epithelial cells *in vivo*, and it infected only CD45^+^ cells (Fig. S7J and K).

The transcription of the six structural genes of NDV by viral RNA-directed RNA polymerase L follows the canonical stop-start mechanism of paramyxoviruses and other nonsegmented negative-strand RNA viruses (23, 24). The guide RNA genes were separated by short intercistronic nucleotide sequences. Transcription takes place within the ribonucleoprotein core structure. Transcription starts at the 3′ end, with viral RNA-dependent RNA polymerase L processing in the 5′-to-3′ direction, and it terminates at the transcription termination signal of each gene after polyadenylation. Polymerase L either falls off the genomic RNA template or reinitiates the transcription of the next gene. mRNA copy numbers depend on the reinitiation of transcription (Fig. 7G). Nucleoprotein (NP) was transcribed first and at the highest level, proceeding down the genome to L last and at the lowest level. When we quantified the relative expression levels of NDV structural genes as a function of the viral load, we observed that the gene expression distribution roughly matched the expected pattern in all cells and the identified fibroblast, endothelial, epithelial, myeloid, and T cells, with the most transcripts being derived from NP and the fewest being derived from the 5′ genes P, M, F, HN, and L (Fig. 7H).

Next, we exploited the host gene expression changes between infected and bystander cells. Because ISGs were typically associated with viral RNA sensing, we next examined the expression of ISGs in these cells. Compared with matched cell types, ISGs exhibited elevated expression levels in these virus-positive cells compared with the virus-negative cells. In all cells, RSAD2, IFIH1, TRANK1, DDX27, and AVD were upregulated, and TGM2 was downregulated (Fig. 7I). In endothelial cells, STAT1, TRIM25, RSAD2, IFIH1, TRANK1, and AVD were upregulated, and LYG2 was downregulated (Fig. 7I). In fibroblast cells, only two ISGs were changed, with DDX27 being upregulated and TGM2 being downregulated (Fig. 7I). In myeloid cells, STAT1, TRIM25, RSAD2, and IFIH1 were upregulated, and ASS1 and CTSS were downregulated (Fig. 7I). In epithelial cells, STAT1, TRIM25, RSAD2, IFIH1, TRANK1, and AVD were upregulated (Fig. 7I). In T cells, STAT1, TRIM25, RSAD2, and IFIH1 were upregulated (Fig. 7I). ISG expression was not significantly changed in epithelial and T cells.

Pathway analysis of DEGs using GO and KEGG terms revealed the response to virus for DEGs in virus-positive and virus-negative cells, and most gene functions were enriched in the defense response to virus, the innate immune response, and the inflammatory response and were involved in autophagy, apoptosis, and the Jak-STAT pathway (Fig. 7J and K).

Usually, the IFN signal of total cells was increased compared with virus-positive and virus-negative cells, including endothelial, epithelial, myeloid, and T cells, although the IFN response was weak in fibroblast cells. These results suggest that endothelial, epithelial, myeloid, and T cells were the main cell types interacting with NDV particles and accounted for the highest procurement of viral material, in line with their role as the first innate immune responders to infection.

### Cell-cell communication to identify the ligand-receptor pair among the control, Herts/33, and LaSota groups.

**FIG 8:**
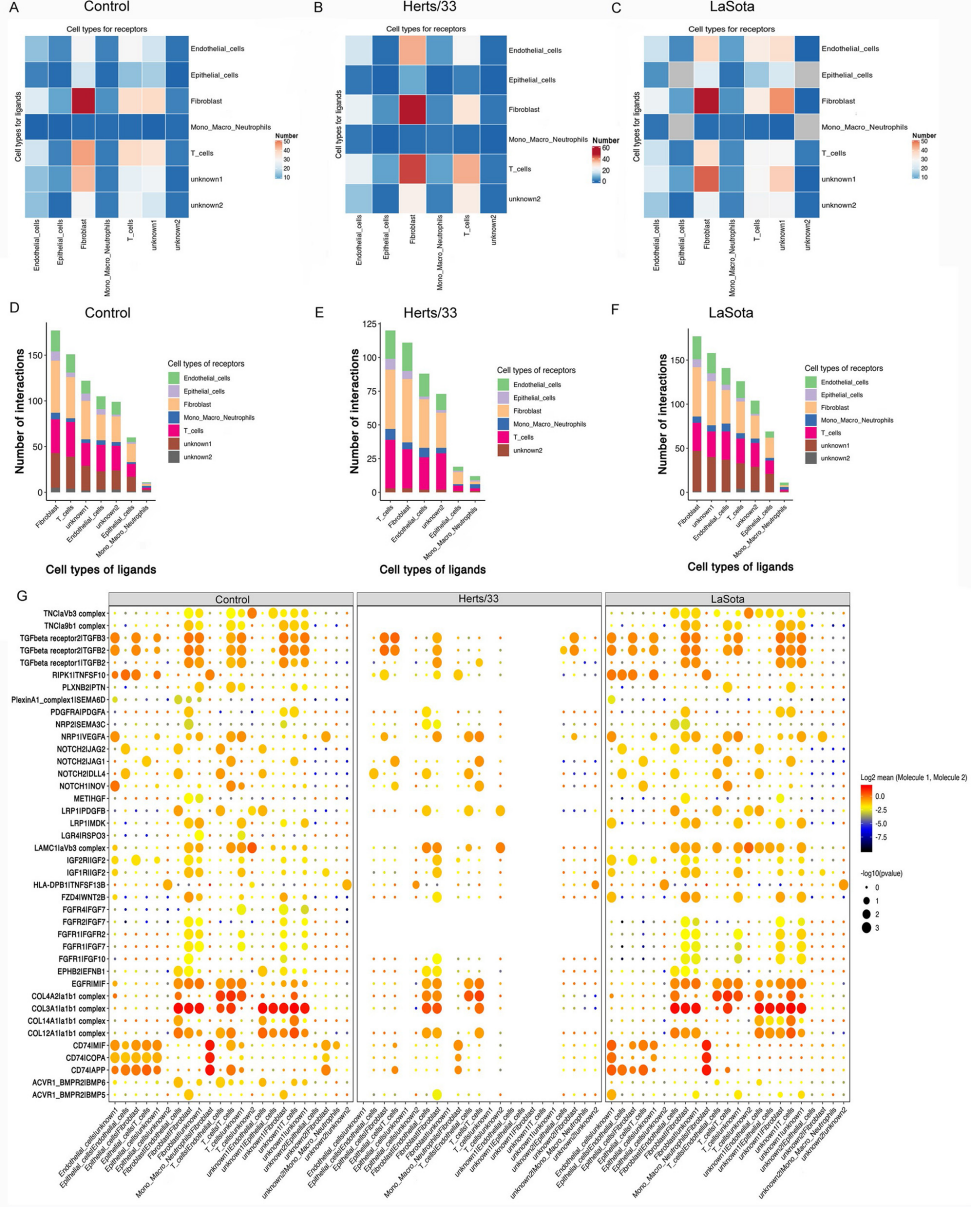
Cell-cell communication. (A to C) Numbers of predicted interactions between mono-macro-neutrophil, T, fibroblast, endothelial, epithelial, unknown1, and unknown2 cells based on CellPhoneDB in the control (A), Herts/33 (B), and LaSota (C) groups (*P ≤ *0.05). (D to F) Numbers of ligand-receptor pairs interacting between cell types in the control (D), Herts/33 (E), and LaSota (F) groups (*P ≤ *0.05). (G) Predicted pairwise interactions between cells, compared with the control, Herts/33, and LaSota groups. The left side of the vertical line represents the ligand, and the right side of the vertical line represents the receptor. Circle sizes represent significance levels as log_10_ *P* values. Color bars from blue to red represent the normalized expression values of both the ligands and receptors.

Since our data revealed NDV target cells and their response after NDV infection, we explored the predicted interactome between these cell types to gain refined insights (Fig. S8). First, we calculated the interactions between cell types (*P ≤* 0.05) separately for the virulent Herts/33 strain and the nonvirulent LaSota strain of NDV. We then assessed the differences in the number of specific interactions. After NDV infection, the receptor-ligand interactions showed different patterns between the Herts/33 and LaSota groups. In the control group, the interactions occurred in all cell types, including fibroblast, endothelial, epithelial, mono-macro-neutrophil, T, unknown1, and unknown2 cells (Fig. 8A and D). The number of interactions in fibroblast cells was the highest, and the number gradually decreased in T, unknown1, endothelial, unknown2, epithelial, and mono-macro-neutrophil cells (Fig. 8A and D). However, interactions after Herts/33 infection were found in fibroblast, endothelial, epithelial, myeloid, T, and unknown2 cells but not unknown1 cells (Fig. 8B and E). The number of interactions in T cells was the highest, and the number gradually decreased in fibroblast, endothelial, epithelial, unknown2, epithelial, and mono-macro-neutrophil cells (Fig. 8B and E). In the LaSota group, the interactions occurred in all cell types, including fibroblast, endothelial, epithelial, myeloid, T, unknown1, and unknown2 cells (Fig. 8C and F). The number of interactions in fibroblast cells was the highest, and the number gradually decreased in unknown1, endothelial, unknown2, epithelial, and mono-macro-neutrophil cells (Fig. 8C and F).

The specific interactions between receptors and ligands among the control, highly virulent NDV Herts/33 strain, and nonvirulent LaSota strain groups showed different patterns, although the specific interaction patterns between the control and nonvirulent LaSota strain groups showed slightly similar scenarios (Fig. 8G). In the control group, the collagen-integrin complex interaction pair (COL4A2/α1β1, COL3A1/α1β1, COL14A1/α1β1, or COL12A1/α1β1) was the most prominent. Specifically, the COL4A2/α1β1 pair was the most prominent in T cells to endothelial cells, T cells to T cells, and T cells to unknown1 cells; COL3A1/α1β1 was the most prominent in fibroblast cells to endothelial cells, fibroblast cells to unknown1 cells, fibroblast cells to fibroblast cells, T cells to T cells, unknown1 cells to endothelial cells, unknown1 cells to epithelial cells, unknown1 cells to fibroblast cells, unknown1 cells to T cells, and unknown1 cells to unknown1 cells. CD74 was found to be highly involved in interactions by binding secreted proteins and cell surface receptors such as migration inhibitory factor (MIF), amyloid beta precursor protein (APP), and COPI coat complex subunit alpha (COPA) between mono-macro-neutrophil and fibroblast cells. In addition, the LAMC1/αvβ3 interaction was highly expressed in T and unknown2 cells (Fig. 8G). No specific interactions were observed in unknown1 cells. These results were consistent the above-described previous results (Fig. 8B and E). No interactions were detected between tenascin C (TNC) and integrin, PLXNB2 and PTN, PlexinA1_complex1 and SEMA6D, PDGFRA and PDGFA, LRP1 and MDK, LGR4 and RSPO3, FGF and FGFRs, COL14A1 and α1β1, and ACVR1_BMPR2 and BMP6 after Herts/33 infection. The LAMC1/αvβ3 interaction between T cells and unknown2 cells was weaker in the Herts/33 group than in the control group. In addition, the interaction of CD74 with MIF, APP, or COPA was weaker in the Herts/33 group than in the control group. In the LaSota group, the FGFR4/FGF7 interaction was not detected in all cells. The LAMC1/αvβ3 interaction between T cells and unknown2 cells was weaker in the LaSota group than in the control group. In addition to the interactions between mono-macro-neutrophil and fibroblast cells, the interaction of CD74 with cell surface receptors, including MIF, APP, and COPA, was stronger between endothelial and unknown1 cells.

## DISCUSSION

Besides being a poultry animal, the chicken is an important model organism and a useful tool for studies of developmental biology and immunology (25, 26). Thus far, single-cell atlases in human and mouse lungs have been developed, and specific cell types in the lungs of humans and mice have been reported (27–29). However, the cell types in the chicken lung remain unexplored. In this study, we characterized the NDV target cell types in the chicken lung at the single-cell transcriptome level and classified cells into five known and two unknown cell types based on human and mouse cell atlas data considering that chicken cells are highly conserved and similar to human and mouse cells. The five known cell types, including fibroblast cells, mono-macro-neutrophils, endothelial cells, epithelial cells, and T cells, have been characterized and identified in humans and mice. These identified cell types have distinct gene signatures. Taking a step further to group cells in a region-specific context, we classified the five known cell types into different clusters with distinct gene signatures. These distinct clusters of cell types were verified to show different functions in viral infection.

Using scRNA-seq data derived from the chicken lung and the chicken embryo fibroblast cell line DF-1, we developed a single-cell transcriptome atlas of NDV in chickens both *in vitro* and *in vivo* by comparing the highly virulent NDV Herts/33 strain and the nonvirulent LaSota strain. Furthermore, we constructed putative trajectories of NDV infection to model gene expression changes along them. In the putative trajectories of NDV infection, we distinguished different paths of infection *in vivo* and *in vitro* or between the virulent Herts/33 strain and the nonvirulent LaSota strain. The gene expression patterns and IFN responses in different putative trajectories were demonstrated. These results can be helpful for understanding NDV infection *in vivo* or *in vitro* and comparing virulent and nonvirulent strains. Interestingly, in endothelial and fibroblast cells, the genes with modeled expression changes along the putative trajectories were enriched in viral budding by the host endosomal sorting complex required for transport (ESCRT) complex. Enveloped viruses escape infected cells by budding through limiting membranes. The ESCRT system, which is classically associated with the sorting and degradation of surface proteins, is a host machinery hijacked by viruses across diverse families (30). NDV relies on the host ESCRT machinery during virus exit, and the budding of NDV depends on an FPIV-like motif in the M protein (31, 32). Thus, endothelial and fibroblast cells could be prior replication sites. Innate immunity is the first line of defense against pathogen infection (33). ISGs are activated during this response and play an important role in antiviral activity (34). We analyzed the IFN response in the different putative trajectories of the identified cells and found that it significantly increased over pseudotime *in vivo*, especially in myeloid and endothelial cells. Recently, Deepak Kaushal et al. found that IFN responsive macrophage populations upregulated ACE2 expression and were infected by severe acute respiratory syndrome coronavirus 2 (SARS-CoV-2) (35). Further analysis of upregulated genes in the macrophages reveal IFN-driven innate antiviral defense and negative regulation of viral genome replication, suggesting a prominent role of macrophage-driven innate immunity in the resolution of SARS-CoV-2 infection. Besides, there is a growing body of literature that demonstrates that myeloid cells can play critical regulatory or immunosuppressive roles, especially following virus elimination (36–38). Therefore, our result suggests that myeloid cell IFN responses correlate with NDV clearance.

The family *Paramyxoviridae* belongs to the order *Mononegavirales*, which contains a large number of vertebrate viruses, some of which cause human diseases such as mumps, measles, and respiratory infections (39). Paramyxoviruses possess single-stranded, nonsegmented, negative-sense RNA genomes of typically 15,000 to 19,000 nucleotides. The processes of transcription and replication are similar in members of the order *Mononegavirales* and follow stop-start transcription, producing a transcriptional gradient, with larger quantities of mRNA being produced by genes closer to the 3′ end of the genome (23, 24). Wignall-Fleming et al. developed a high-throughput sequencing workflow for investigating paramyxovirus transcription and replication, including parainfluenza virus type 2 (PIV2), PIV3, PIV5, and mumps virus (40). The vmRNA abundance gradients differed significantly in all four viruses, but for each virus, the pattern remained relatively consistent with the theoretical gradient throughout infection. We found that the vmRNA abundance of the highly virulent Herts/33 strain in the five cell types was roughly in agreement with the theoretical gradient at the single-cell level. However, we assayed the M gene in only two cell types for the nonvirulent LaSota strain. M proteins were key and central to paramyxovirus particle formation. For many paramyxoviruses, efficient paramyxovirus particle formation can occur only in the presence of a threshold level of functioning M protein (32). M proteins expressed in the absence of the other viral proteins were sufficient for the release of virus-like particles (VLPs) from transfected cells. In NDV, M proteins are needed for efficient virus-like particle production (41). Wise et al. found that the M gene was substantially more sensitive than the F gene in a real-time reverse transcription-PCR assay of NDV clinical samples (42). Therefore, the detection of M genes indicated that the nonvirulent strain infected chickens and suggested that the nonvirulent LaSota strain infected our chicks. However, it was unclear why other genes of the nonvirulent strain of NDV were not detected in clinical samples. This was probably because of the low infection efficiency of the nonvirulent strain by the ocular and nasal routes.

A key element in viral infection is virus entry into host cells. The target cells of NDV have been unclear until now. Our findings indicated that viral infection *in vivo* was highly prevalent in many cell types such as fibroblast, mono-macro-neutrophil, endothelial, epithelial, and T cells because vmRNA was detected in these five cell types. We distinguished virus-infected and noninfected cells and compared the IFN responses among these cells. Several key ISGs were elevated in the virus-positive cells, and these ISGs play antiviral roles in paramyxovirus infection. There is accumulating evidence that DEAD box helicases are involved in the recognition of foreign nucleic acids and the modification of viral infection (43). IFIH1, also known as MDA5, is an IFN-inducible host cell DExD/H box helicase that has been implicated in viral double-stranded RNA (dsRNA) recognition and is critical for detection and IFN production (44, 45). A previous study indicated that the influenza A virus polymerase acidic protein from H3N2 (Wyoming 2003) in A549 cells can interact with DDX27 and induce elevated IFN responses (46). RSAD2 restricts the release of measles virus from infected cells (47). Chicken avidin is a biotin-binding protein expressed under inflammation in several chicken tissues and in the oviduct after progesterone induction (48). Avidin can be induced in chicken embryo fibroblasts by viral transformation and cell damage (49). Irudayam et al. indicated that TRANK1 is an ISG that is induced after hepatitis C virus infection (50). In our study, the levels of IFIH1, DDX27, RSAD2, and TRANK1 were elevated in endothelial, myeloid, epithelial, and T cells. This suggests that these ISGs were the key molecules after NDV infection. In addition, combined with the IFN responses in different putative trajectories, fibroblast, endothelial, myeloid, and T cells were the main cell types interacting with NDV particles and accounted for the greatest procurement of viral material, in line with their role as the first innate immune responders to infection.

Viral infection spreads by overcoming various barriers and is based on the ability of the virus to travel from cell to cell, tissue to tissue, person to person, and even between species. Although there were fundamental differences between these types of propagation, the ability of viruses to exploit and manipulate cell-cell communication contributes to the success of viral infections (51). The extracellular matrix (ECM) of the host cell often functions as a viral barrier. However, some viruses have evolved strategies to bind to specific ECM components (52). This leads to the accumulation of many viral particles on the surface of the host cell, which facilitates effective engagement with the transmembrane receptor(s) or complex host cell receptor(s). Therefore, binding to ECM proteins is an important event in which viruses recognize cell surface molecules and initiate viral infection, and thus, ECM proteins represent the key determinants of viral correlation and infectivity. The ECM is a three-dimensional network of macromolecules, including laminin, fibronectin, collagen, elastin, heparan sulfate, chondroitin sulfate, keratin, and hyaluronic acid, which provide the environment and structure for biochemical support. Collagen-binding integrins are vertebrate transmembrane receptors that interact directly with collagen (53). They are involved in wound healing and fibrosis, but they also contribute to disease progression in various disorders, including inflammatory disorders and tumor angiogenesis. Collagen-binding integrins include α1β1, α2β1, α10β1, and α11β1 (54). Integrins α1β1 and α2β1 may also play important roles in innate immunity and autoimmunity (55). Several studies have suggested that Ross River virus uses integrin α1β1 as a cellular receptor (56). Integrins α1β1 and α2β1 are receptors for the rotavirus enterotoxin (57). The collagen-binding integrin VLA-1 regulates CD8 T cell-mediated immune protection against heterologous influenza virus infection (58). Collagen IV (COL4A1 and COL4A2), a component of the viral biofilm, is induced by the human T cell leukemia virus type 1 (HTLV-1) oncoprotein Tax and impacts virus transmission (59). In this study, the collagen/integrin complex interaction pair (COL4A2/α1β1, COL3A1/α1β1, COL14A1/α1β1, and COL12A1/α1β1) showed the most prominent changes after NDV infection. Thus, the collagen/integrin complex could be a new cell surface receptor for NDV.

Although binding factors and functional receptors have been identified for viruses, such as transferrin receptor for canine and feline parvoviruses and several receptors and attachment factors for adeno-associated virus, the attachment receptor (complex) involved in NDV ECM and cell membrane recognition is still unknown (60–63). Laminin was previously described to interact with sialic acid (64, 65). Laminin cellular receptors include various integrins (e.g., α1β1, α2β1, α3β1, α6β1, α6β4, α7β1, α9β1, and αvβ3), dystroglycan, galectins, heparan sulfate proteoglycans (perlecan and agrin), carbohydrate adduct of proteins 1, Lutheran glycoprotein, syndecans, the 67-kDa laminin receptor, and sulfated glycolipids (52). Different laminin isoforms bind specifically to these molecules, with various affinities, forming distinct complexes. Laminin binds to cells and supports and maintains tissues. LAMC1 encodes the γ1-chain of laminin. Kulkarni et al. identified LAMC1 as a crucial modulator of H-1 parvovirus infection, and oncolytic H-1 parvovirus binds to sialic acid on laminins for cell attachment and entry (66). By modulating multiple signaling pathways, laminins influence basic cellular processes such as adhesion, proliferation, migration, differentiation, and tumor metastasis. In this study, we found that the LAMC1/αvβ3 interaction between T cells and unknown2 cells was weaker after NDV infection. The cell surface binding and entry of NDV are dependent on sialic acid (67, 68). Given these results, LAMC1 could be a key determinant of NDV infection and an important player in the NDV life cycle as a cell attachment factor.

CD74 (the MHC class II-associated invariant chain) is a homotrimeric membrane protein that acts as a receptor for macrophage MIF (69). MIF plays a role in innate immunity and subsequent adaptive responses (70). Recent studies have indicated that the CD74 protein can inhibit Ebola virus and severe acute respiratory syndrome coronavirus 2 (SARS-CoV-2) infections at the same time (71). Other studies have identified a major role for CD74-dependent cross-priming in the generation of responses to viral and cell-associated antigens (72). In our study, CD74 was involved in NDV infection, particularly in the virulent Herts/33 strain. Therefore, CD74 could be an important attachment factor for the virulent NDV Herts/33 strain. The collagen/integrin complex, LAMC1, and CD74 could be intervention targets for NDV.

In summary, our study established the single-cell transcriptome landscape for NDV at single-cell resolution both *in vitro* and *in vivo*. We identified five distinct cell types in the chicken lung and constructed putative trajectories of NDV infection to model gene expression changes along them. The heterogeneity of bystander cells compared with cells infected with NDV was revealed by measuring intracellular viral RNA. The potential NDV cell surface receptor and ligand have been uncovered and can help in interventions against NDV. Our results open the way to interventions specifically targeting infected cells, suggest principles of virus-host interactions applicable to NDV and other similar pathogens, and highlight the potential of simultaneous single-cell measurements of both host and viral transcriptomes for delineating a comprehensive map of infection *in vitro* and *in vivo*. Thus, this study can be a useful resource for the further investigation and understanding of NDV. CD45 is an immune cell marker. In this study, we used CD45 marker gene to distinguish the immune cells and non-immune cells. In this study, we have constructed the single cell atlas both *in vitro* and *in vivo*. We will investigate the immnune response of lung after NDV infection further. This will be helpful the understanding the immune response after NDV infection and be benefit to the design of vaccine.

## MATERIALS AND METHODS

### Animals and ethics statement.

SPF chicken eggs were purchased from the Beijing Boehringer Ingelheim Merial Vital Laboratory Animal Technology Co., Ltd., China. The operation and treatment of animals were approved by the Institutional Animal Care and Use Committee of the Shanghai Veterinary Research Institute, Chinese Academy of Agricultural Sciences (SV-20201225-Y02).

After SPF chicks hatched, they were housed in separate negative-pressure isolators in biosafety level 2 facilities and supplied with feed and water *ad libitum*. Three-day-old SPF chicks (*n *= 21) were randomly distributed into three groups, and each group contained seven chicks. One group (*n *= 7) was challenged with the LaSota lentogenic strain of NDV (200 μL of 10^8^ 50% egg infectious doses [EID_50_]) by the ocular and nasal routes (50 μL into each eye and nostril), whereas the other group (*n *= 7) was challenged with the highly virulent Herts/33 strain of NDV (200 μL of 10^3^ EID_50_). The control group (*n *= 7) was treated with 200 μL of phosphate-buffered saline (PBS) by the same routes. Chicks were sacrificed after 3 days of treatment. Finally, two chicks in each group were used for single-cell sorting.

### Single-cell sorting.

Infected or PBS-treated chickens were sacrificed by spinal cord injury. Their lungs were subsequently perfused with PBS through the right ventricle and divided into two groups that were subjected to different dissociation techniques. One-half of the entire pool of lungs was dissected and dissociated into single-cell suspensions using multitissue dissociation kit 1 (Miltenyi Biotec) in combination with a gentle magnetically activated cell sorting (MACS) dissociator (Miltenyi Biotec) and enzymatic dissociation. The other half of the lung pool was minced on ice to <1-mm^3^ pieces, suspended in 5 mL of digestion buffer consisting of elastase (3 U/mL; Worthington Biochemical Corporation) and DNase I (0.33 U/mL; Sigma-Aldrich) in Dulbecco’s modified Eagle’s medium (DMEM)–F-12 medium, incubated with frequent agitation at 37°C for 20 min, and briefly triturated (73). Next, an equal volume of a mixture DMEM–F-12 medium supplemented with 10% fetal bovine serum (FBS) and penicillin-streptomycin (1 U/mL; Biological Industries) was added to the single-cell suspensions in both groups. Following enzymatic incubation, cells derived from the same lungs (after the two dissociation techniques) were passed through a 100-μm sieve and then through a 70-μm sieve, pelleted again (300 × *g* for 5 min at 4°C), and resuspended in MACS buffer (0.5% bovine serum albumin and 2 mM EDTA in PBS). Red blood cells were removed by depletion with chicken red blood antibody (Fitzgerald Industries International, Inc.) against fluorescein isothiocyanate (FITC) conjugated to magnetic beads (Miltenyi Biotec). Cell populations were sorted using chicken CD45 antibody (Southern Biotech) against biotin conjugated to magnetic beads (Miltenyi Biotec).

Cells of the chicken embryo fibroblast cell line DF-1 (ATCC, CRL-12203) were seeded into a 15-cm plate and cultured overnight. When the cell density reached approximately 80% confluence, cells were infected with the highly virulent Herts/33 strain at a multiplicity of infection (MOI) of 1 and incubated at 37°C with 5% CO_2_ for 1 h. The growth medium was replaced with DMEM supplemented with 2% FBS, and the mixture was incubated for 12 h before harvest. There was an obvious cytopathic effect on DF-1 cells, which confirmed NDV infection. Uninfected cells were considered negative controls. Single-cell suspensions were prepared based on 10× Genomics protocols.

### Sample preparation protocol.

Cells isolated from each group were captured in droplet emulsions using the 10× Genomics chromium single-cell instrument, and libraries were prepared using the 10× Genomics 3′ single-cell v2 protocol for the DF-1 cell line group and the 10× Genomics 3′ single-cell v3 protocol for the chicken group as 10× Genomics described. All 10× libraries were pooled and sequenced on the NovaSeq 6000 platform (Illumina).

### scRNA-seq data processing.

The raw deep-sequencing data were processed using the 10× Genomics software package CellRanger (v5.0.0). The reads were aligned to a concatenation of the chicken reference genome (GRCg6a) and the Newcastle disease virus (NDV) genome. The complete genome sequence of the highly virulent Herts/33 strain was obtained from GenBank accession number AY741404.1, and that of the lentogenic strain LaSota was obtained from GenBank accession number JF950510.1. The chicken transcriptome was generated by filtering the GRCg6a genome assembly for protein-coding genes defined in the GTF file.

We processed the unique molecular identifier (UMI) count matrix using the Seurat (v3.1.1) R package (74). To remove low-quality cells and likely multiplet captures, a major concern in microdroplet-based experiments, we applied a criterion to filter out cells with a UMI/gene number of the limit of the mean value ± 2 standard deviations with a Gaussian distribution of the UMI/gene numbers in each cell. Following visual inspection of the distribution of cells by the fraction of mitochondrial genes expressed, we further discarded low-quality cells where >30% of the counts belonged to mitochondrial genes. In addition, we used the DoubletFinder package (v2.0.2) (75) to identify potential doublets. After applying these quality control criteria, 91,447 single cells were included in the downstream analyses. Library size normalization was performed using the NormalizeData function in Seurat to obtain the normalized count. Specifically, the global-scaling normalization method LogNormalize was used to normalize the gene expression measurements for each cell by the total expression level, multiplied by a scaling factor (10,000 by default), and the results were log transformed.

The top variable genes across single cells were identified using a method described previously by Macosko et al. (76). The most variable genes were selected using the FindVariableGenes function (mean.function = FastExpMean, dispersion.function = FastLogVMR) in Seurat. To remove the batch effects in the scRNA-seq data, a mutual nearest-neighbor method described previously by Haghverdi et al. was performed using the batchelor R package (77). Graph-based clustering was performed to cluster cells according to their gene expression profiles using the FindClusters function in Seurat. Cells were visualized using the two-dimensional *t*-distributed stochastic neighbor embedding (t-SNE) algorithm with the RunTSNE function in Seurat. We used the FindAllMarkers function (test.use = bimod) in Seurat to identify marker genes of each cluster. For a given cluster, FindAllMarkers identified positive markers compared with all of the other cells.

Differentially expressed genes (DEGs) were identified using the FindMarkers function (test.use = MAST) in Seurat. A *P* value of <0.05 and a |log_2_ fold change| of >0.58 were set as the thresholds for significant differential expression. GO and KEGG pathway enrichment analyses of DEGs were performed using R based on the hypergeometric distribution.

### Cell type annotation.

Clustering results for individual or grouped samples were visualized using t-SNE. Cell types were classified based on differential expression analysis with cluster-specific marker genes identified using the FindMarkers function. GO enrichment analysis of cell type-specific marker genes was performed using ToppGene online tools.

### Gene set variation analysis.

To perform gene set variation analysis (GSVA), the GSEABase package (v1.44.0) was used to load the gene set file that was downloaded from the KEGG database (https://www.kegg.jp/). To assign pathway activity estimates to individual cells, GSVA was performed using standard settings, as implemented in the GSVA package (v1.30.0) (78). Differences in the pathway activities scored per cell were calculated using the LIMMA package (v3.38.3).

### Pseudotime analysis.

We determined the putative pseudotime of NDV infection using the Monocle2 package (v2.9.0) (79). The raw count was first converted from the Seurat object to the CellDataSet object using the importCDS function in Monocle. We used the differential GeneTest function of the Monocle2 package to select ordering genes (*q* value of <0.01) that were likely to be informative in the ordering of cells along the pseudotime trajectory. Dimensional reduction clustering analysis was performed using the reduceDimension function, followed by trajectory inference using the orderCells function with default parameters. Gene expression was plotted using the plot_genes_in_pseudotime function to track changes over pseudotime. GO terms of each module were analyzed using the clusterProfiler R package.

### NDV sequence detection.

We annotated the structural genes of NDV, and the virus GTF file with detailed annotation information and the viral genome were combined with the chicken reference genome to construct a new reference genome. The CellRanger pipeline was performed again, and routine quantitative postquality control was performed to obtain standardized expression using this new concatenation of the GRCg6a and NDV genomes. Single cells with the same cellular barcode intersection with the previous results were used for calculating viral gene expression. We inferred the viral load within a cell from the number of UMIs that were aligned with viral segments. Specifically, the viral load was the proportion of viral UMIs (i.e., UMIs aligned with one of the viral segments) of the total UMI content of a given cell. Accordingly, we defined two groups of cells: (i) infected cells detectably expressing viral mRNA (vmRNA) and (ii) bystander cells not detectably expressing vmRNA.

### IFN-stimulated genes induced by NDV infection.

The transcripts in virus-positive or virus-negative cells were queried against the Interferome database (v2.01) (http://interferome.its.monash.edu.au), which contains avian IFN-stimulated genes (ISGs) identified by microarray analysis and RNA-seq data from primary chicken embryo fibroblasts treated with a chicken type I IFN (IFN-α) (80, 81).

### Cell-cell communication analysis.

We used CellPhoneDB (v2.0) to identify biologically relevant ligand-receptor interactions from single-cell transcriptomics (scRNA-seq) data using homologous human genes (82). We defined a ligand or receptor as being “expressed” in a particular cell type if 10% of the cells of that type had nonzero read counts for the ligand-receptor-encoding gene. Statistical significance was assessed by randomly shuffling the cluster labels of all cells and repeating these steps, which generated a null distribution for each ligand-receptor pair in each pairwise comparison of two cell types. After running 1,000 permutations, *P* values were calculated with the normal distribution curve generated from the permuted ligand-receptor pair interaction scores. To define networks of cell-cell communication, we linked any two cell types where the ligand was expressed in the former cell type and the receptor was expressed in the latter. The Igraph and Circlize R packages were used to display the cell-cell communication networks.

### Immunofluorescence.

Immunostaining was performed on 5-μm-thick formalin-fixed, paraffin-embedded lung tissue. Briefly, antigen retrieval was accomplished by microwaving the sections at 98°C in 10 mM Tris-EDTA (pH 8.0). Slides were blocked with 3% methanol-hydrogen peroxide solution at room temperature for 25 min and then washed three times (5 min each time) with PBS (pH 7.4). After blocking with 0.5% BSA for 30 min, the primary antibody was added to the slides for incubation at 4°C overnight. After washing with PBS (pH 7.4), the slides were incubated with polymer horseradish peroxidase-conjugated antibody specific to rabbits. FITC-TSA was added to each slide and incubated at room temperature. Antigen retrieval was performed on stained slides to prepare them for staining to detect the next target protein. Nuclei were counterstained with DAPI. The primary antibodies used were anti-NRP1 (1:50, CY5243; Abways), anti-PDGFR alpha (1:30, CY6714; Abways), anti-CSF1R (1:20, CY1032; Abways), and anti-LEF1 (1:250, CY5350; Abways),which were obtained from Abways.

### Data availability.

Raw sequencing reads from the scRNA-seq experiments generated for this study have been deposited in GEO DataSets. The publicly available data sets that supported this study are available from the GEO. The accession number is PRJNA834764; GEO number is GSE202128.
