# Supplementary material for: Single-Cell Transcriptome Atlas of Newcastle Disease Virus in Chickens Both In Vitro and In Vivo
Source: Microbiol Spectr. 2023 May 16;11(3):e05121-22. doi: 10.1128/spectrum.05121-22 (PMC10269786; doi:10.1128/spectrum.05121-22)
Supplement: Supplemental file 2 — Supplemental material. Download spectrum.05121-22-s0002.pdf, PDF file, 10.1 MB [file spectrum.05121-22-s0002.pdf]

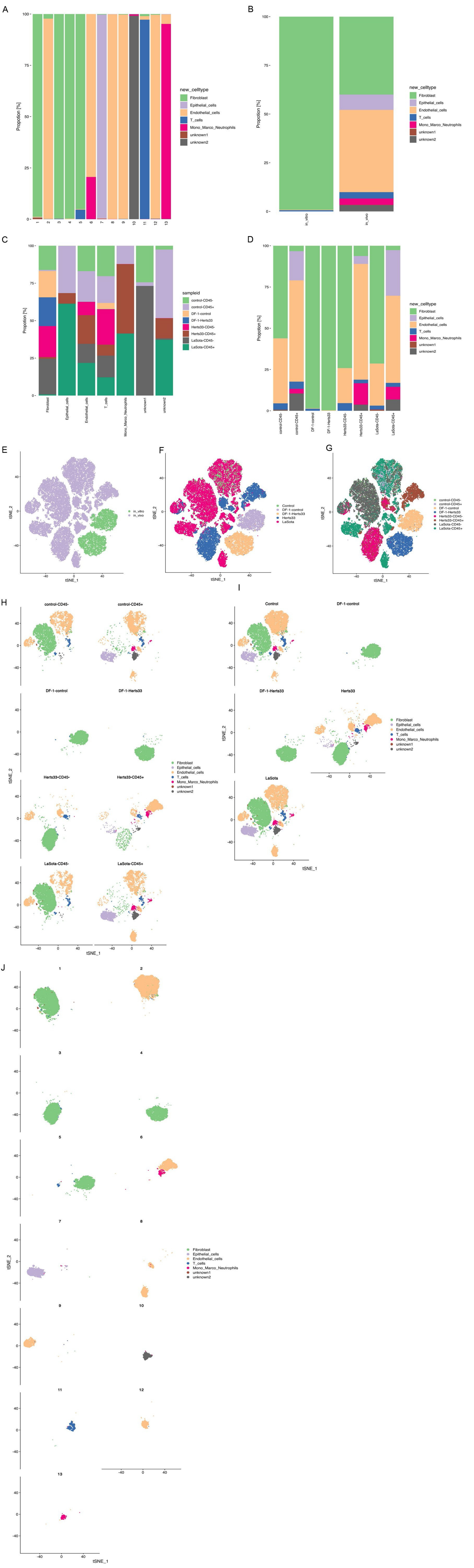

Supplementary FIgure1

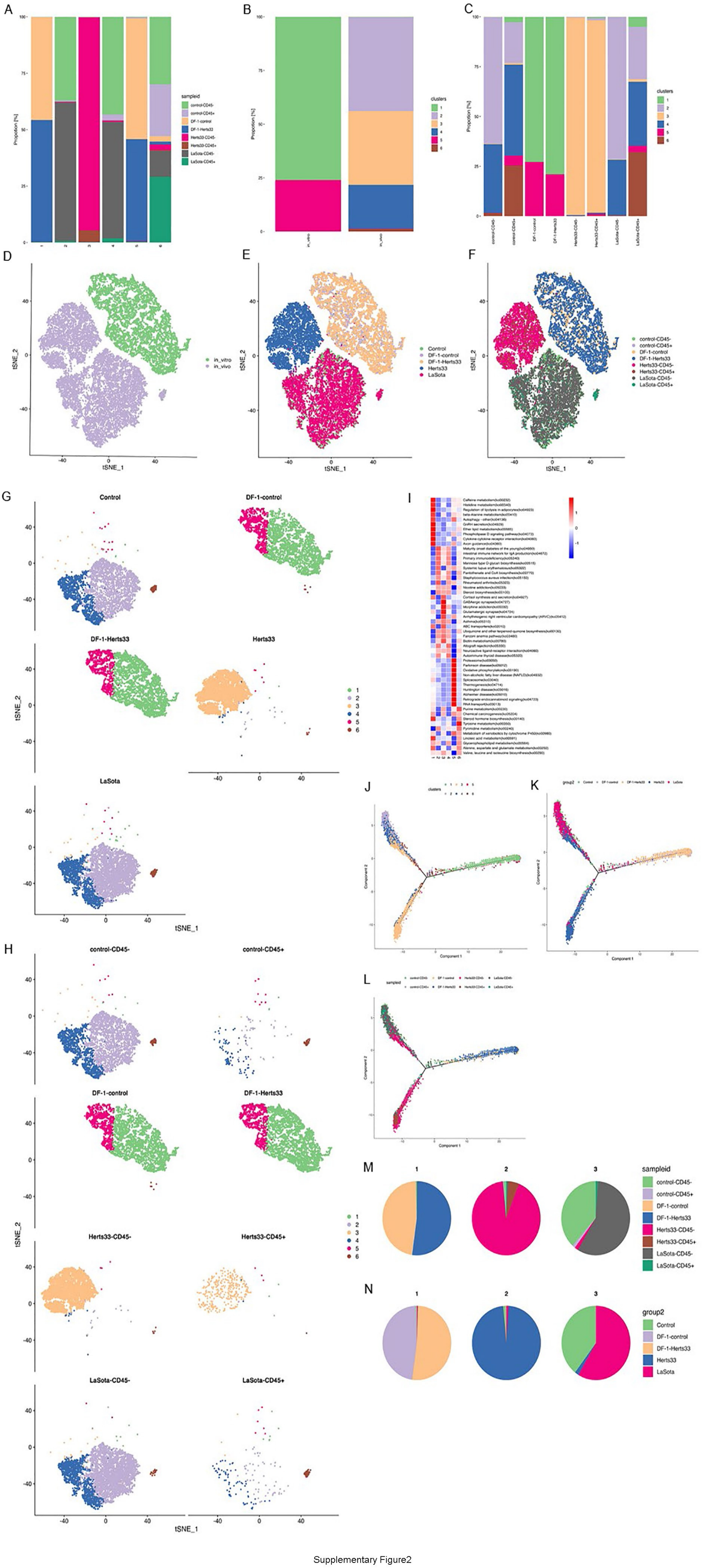

Supplementary Figure2

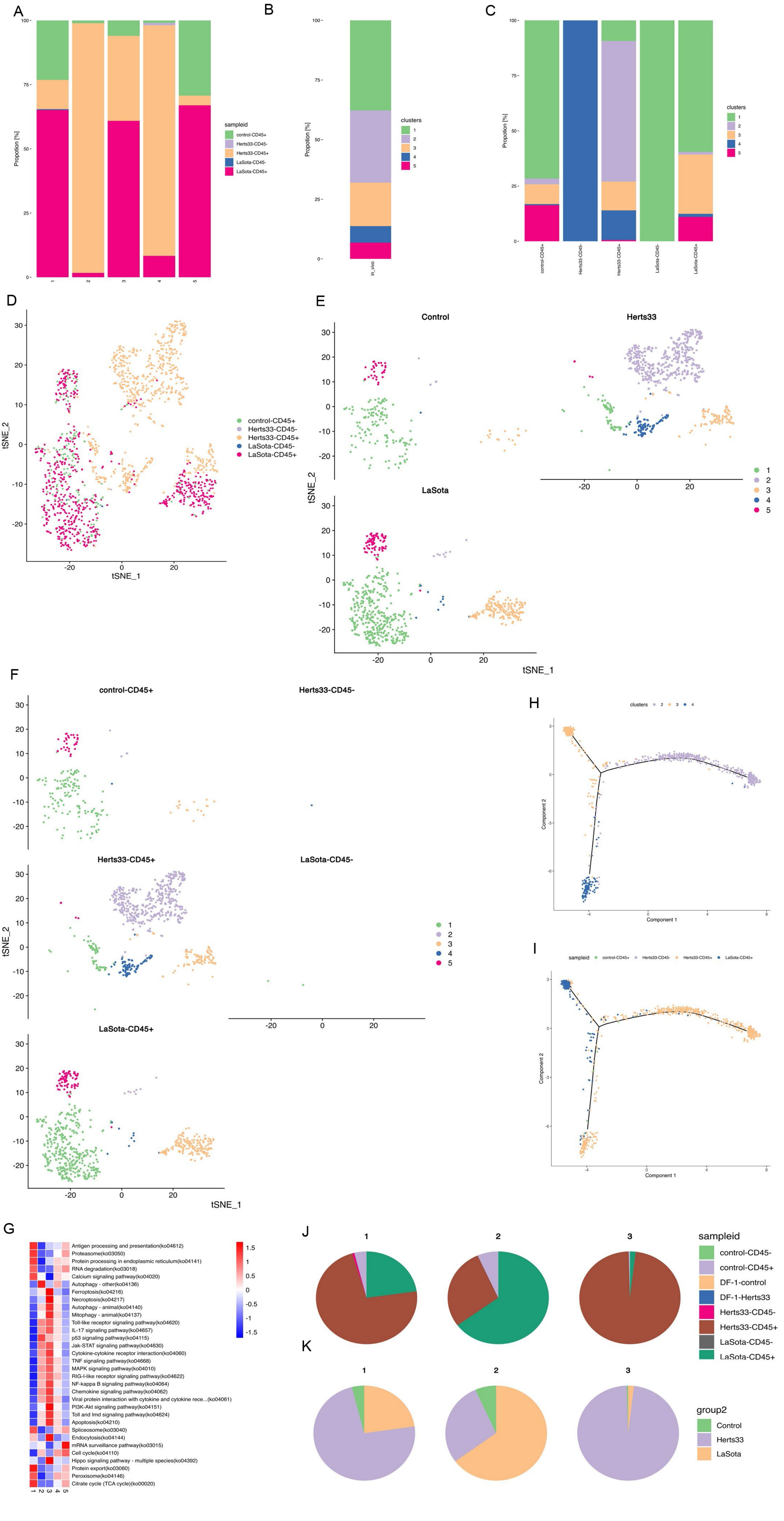

Supplementary Figure3

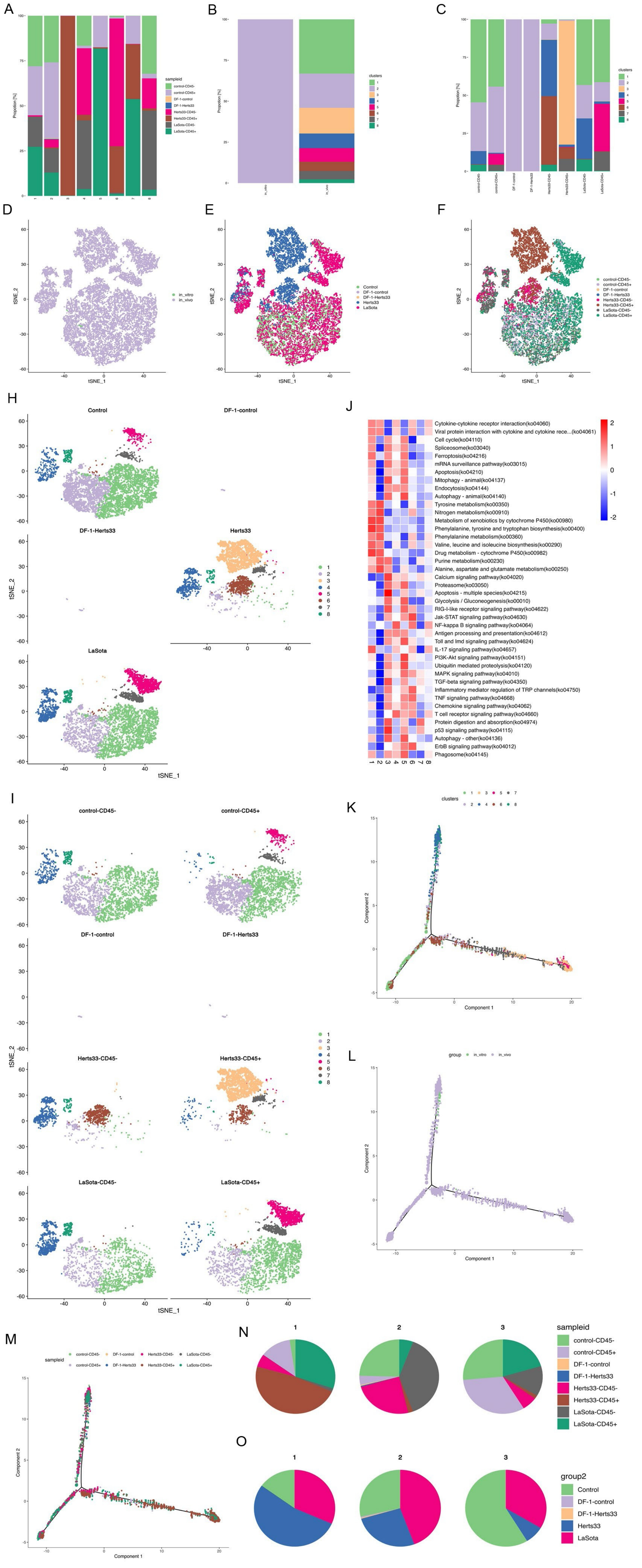

Supplementary Figure4

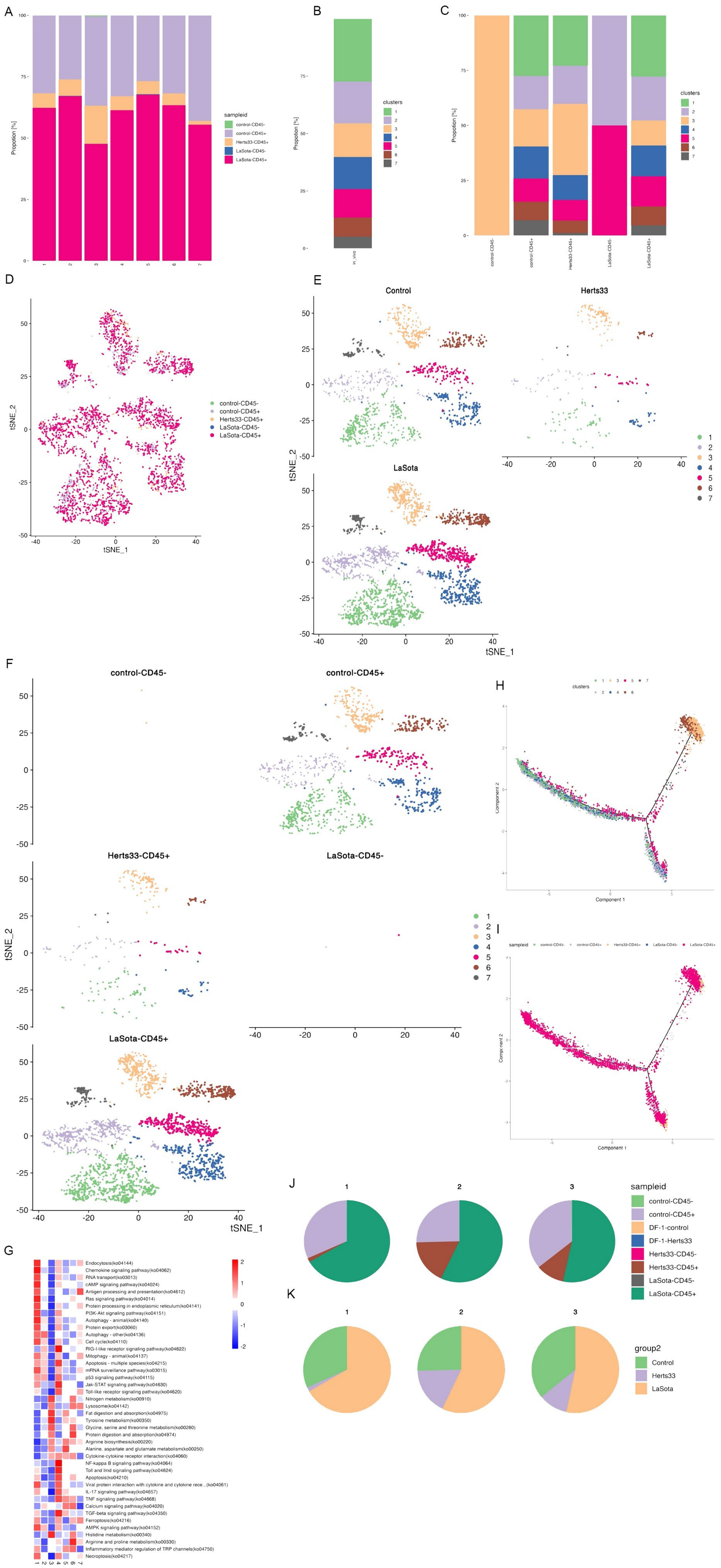

Supplementary Figure5

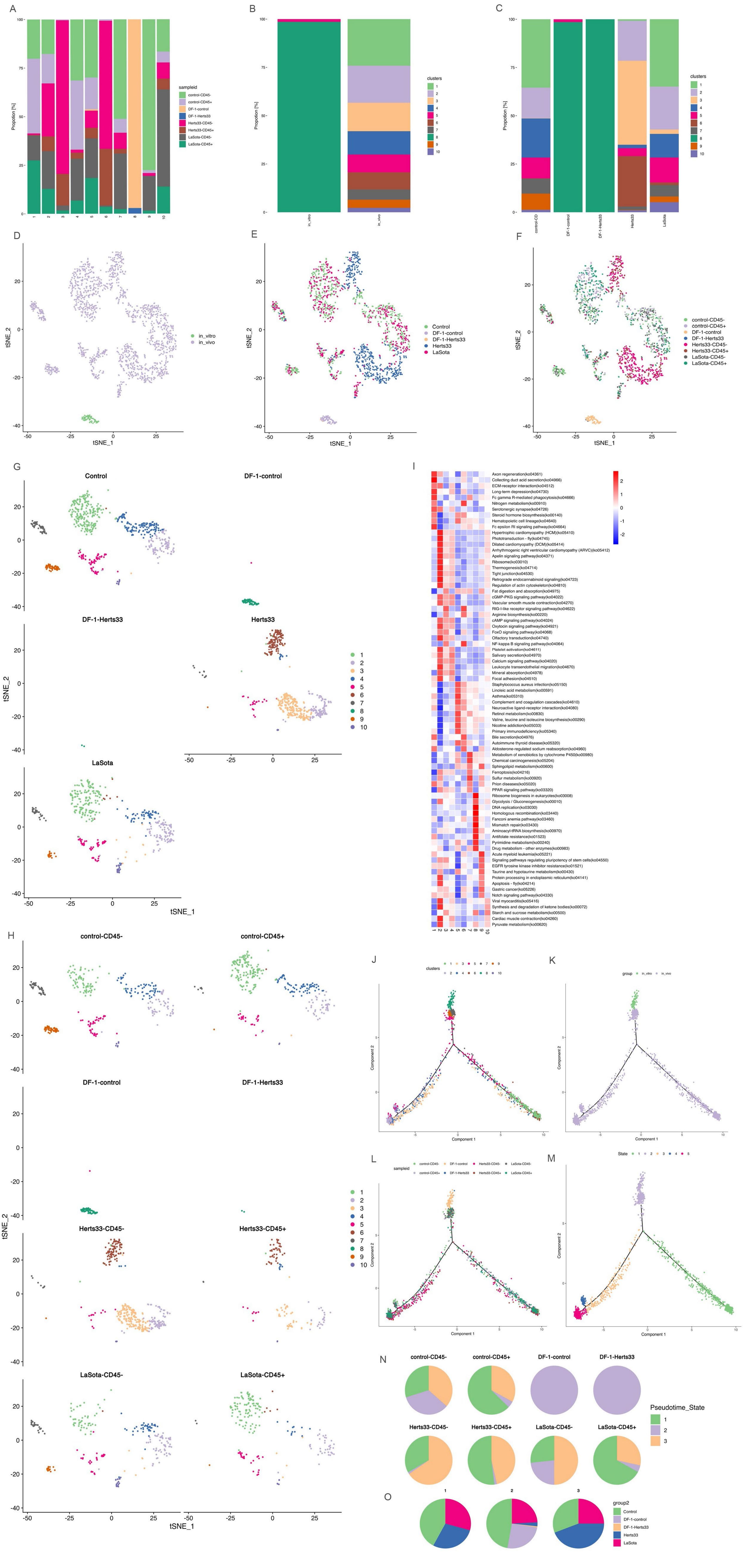

Supplementary Figure6

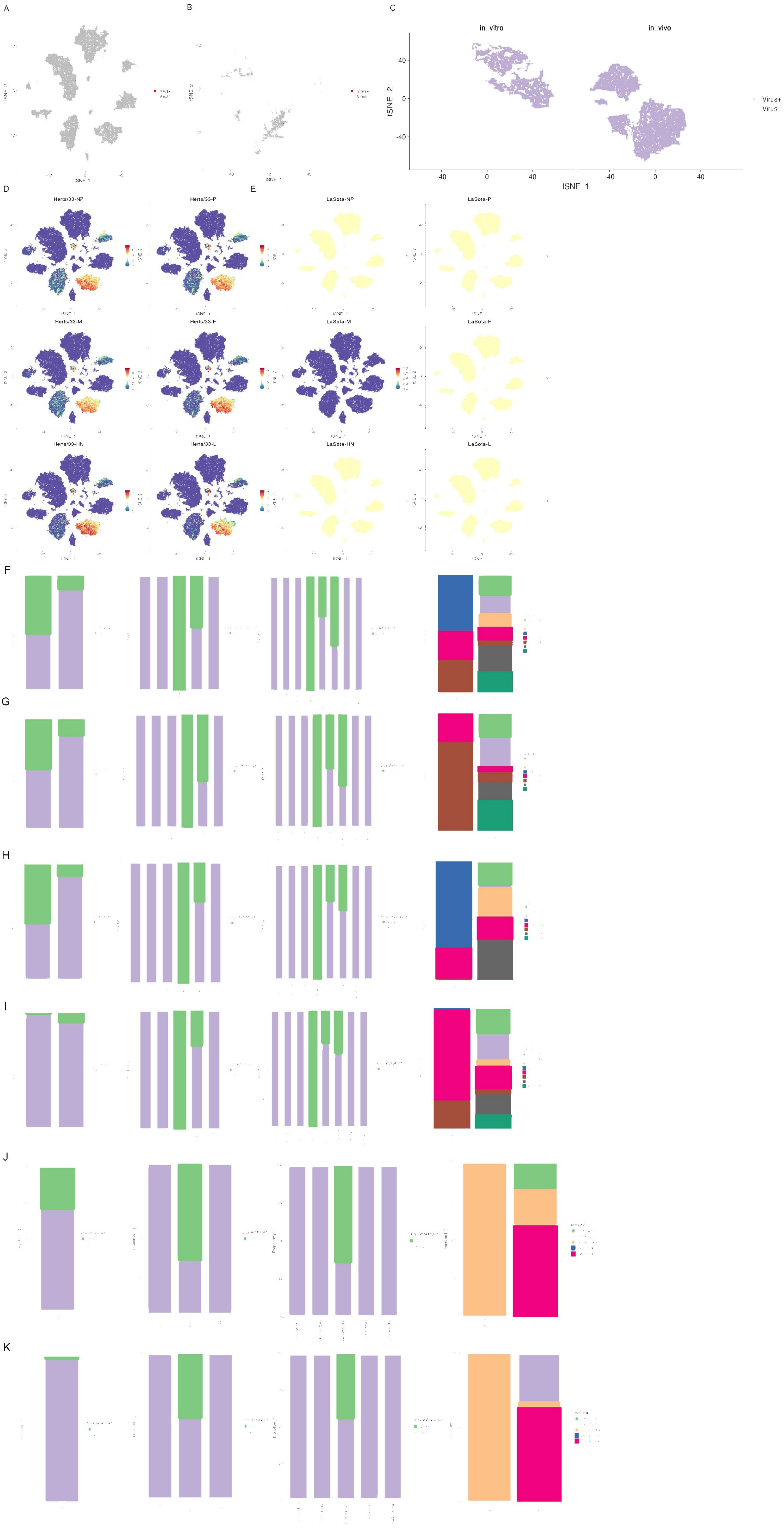

Supplementary Figure7

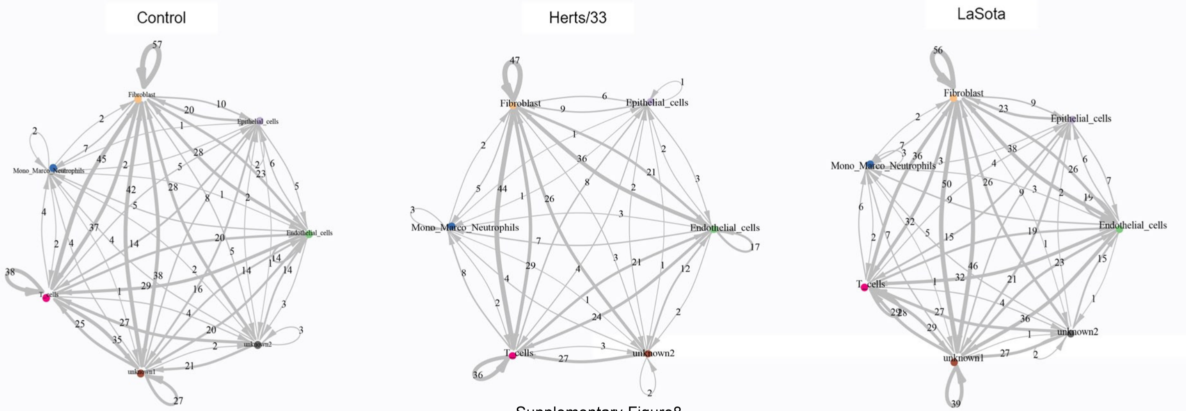

Supplementary Figure8

Supplementary Figure 1. Global single-cell transcriptional landscape in the chicken lung and DF-1 cell line. A. Barplots showing the proportion of each cluster in each cell type. B. Barplots showing the proportion of each cell type both *in vitro* and *in vivo*. C. Barplots showing the proportion of each sample in each cell type. D. Barplots showing the proportion of each cell type in each sample. t-SNE plot of the sample both *in vitro* and *in vivo*. F. t-SNE plot of the sample in control, Herts/33, LaSota, DF-1-control, and DF-1-Herts/33. G. t-SNE plot of the sample in control-CD45$^+$/CD45$^-$, Herts/33-CD45$^+$/CD45$^-$, LaSota-CD45$^+$/CD45$^-$, DF-1-control, and DF-1-Herts/33. H. t-SNE plot of the sample in control-CD45$^+$/CD45$^-$, Herts/33-CD45$^+$/CD45$^-$, LaSota-CD45$^+$/CD45$^-$, DF-1-control, and DF-1-Herts/33. I. t-SNE plot of the sample in control, Herts/33, LaSota, DF-1-control, and DF-1-Herts/33. J. t-SNE plot of each cell type in each cluster.

Supplementary Figure 2. Single-cell profiling of fibroblast cells. A. Barplots showing the proportion of each sample in each cluster. B. Barplots showing the proportion of each cell type both *in vitro* and *in vivo*. C. Barplots showing the proportion of each cluster in each sample. D. t-SNE plot of the sample both *in vitro* and *in vivo*. E. t-SNE plot of the sample in control, Herts/33, LaSota, DF-1-control, and DF-1-Herts/33. F. t-SNE plot of the sample in control-CD45$^+$/CD45$^-$, Herts/33-CD45$^+$/CD45$^-$, LaSota-CD45$^+$/CD45$^-$, DF-1-control, and DF-1-Herts/33. G. t-SNE plot of each cell cluster in control, Herts/33, LaSota, DF-1-control, and DF-1-Herts/33. H. t-SNE plot of each cell cluster in control-CD45$^+$/CD45$^-$, Herts/33-CD45$^+$/CD45$^-$,

LaSota-CD45$^+$/CD45$^-$, DF-1-control, and DF-1-Herts/33. I. Differences in pathway activities scored per cell by GSVA between epithelial cells isolated from different epithelial clusters, with enriched KO terms; $P < 0.05$. J–L. Pseudotime trajectory plot representing the features of each cluster (J) and in each sample (K and L) in the three identified states. M and N. Pie charts showing the proportion of cells in each sample in the three identified states.

Supplementary Figure 3. Single-cell profiling of myeloid cells. A. Barplots showing the proportion of each sample in each cluster. B. Barplots showing the proportion of each cell type *in vivo*. C. Barplots showing the proportion of each cluster in each sample. D. t-SNE plot of the sample in control, Herts/33, LaSota, DF-1-control, and DF-1-Herts/33. E. t-SNE plot of each cell cluster in the control, Herts/33, and LaSota groups. H. t-SNE plot of each cell cluster in control-CD45$^+$, Herts/33-CD45$^+$/CD45$^-$, and LaSota-CD45$^+$/CD45$^-$. G. Differences in pathway activities scored per cell by GSVA between myeloid cells isolated from different epithelial clusters, with enriched KO terms; $P < 0.05$. H and I. Pseudotime trajectory plot representing the features of clusters 2, 3, and 4 (H) and in each sample (I) in the three identified states. J and K. Pie charts showing the proportion of cells in each sample in the three identified states.

Supplementary Figure 4. Single-cell profiling of endothelial cells. A. Barplots showing the proportion of each sample in each cluster. B. Barplots showing the proportion of each cell type both *in vitro* and *in vivo*. C. Barplots showing the

proportion of each cluster in each sample. D. t-SNE plot of the sample both *in vitro* and *in vivo*. E. t-SNE plot of the sample in control, Herts/33, LaSota, DF-1-control, and DF-1-Herts/33. F. t-SNE plot of the sample in control-CD45^{+}/CD45^{-}, Herts/33-CD45^{+}/CD45^{-}, LaSota-CD45^{+}/CD45^{-}, DF-1-control, and DF-1-Herts/33. G. t-SNE plot of each cell cluster in control, Herts/33, LaSota, DF-1-control, and DF-1-Herts/33. H. t-SNE plot of each cell cluster in control-CD45^{+}/CD45^{-}, Herts/33-CD45^{+}/CD45^{-}, LaSota-CD45^{+}/CD45^{-}, DF-1-control, and DF-1-Herts/33. I. Differences in pathway activities scored per cell by GSVA between endothelial cells isolated from different epithelial clusters, with enriched KO terms; $P < 0.05$. J–L. Pseudotime trajectory plot representing the features of each cluster *in vivo* (J), *in vitro* (K), and in each sample (L) in the three identified states. M and N. Pie charts showing the proportion of cells in each sample in the three identified states.

Supplementary Figure 5. Single-cell profiling of epithelial cells. A. Barplots showing the proportion of each sample in each cluster. B. Barplots showing the proportion of each cell type *in vivo*. C. Barplots showing the proportion of each cluster in each sample. D. t-SNE plot of the sample in control-CD45^{-}/control-CD45^{+}, Herts/33-CD45^{+}, and LaSota-CD45^{+}/CD45^{-}. E. t-SNE plot of each cell cluster in the control, Herts/33, and LaSota groups. F. t-SNE plot of each cell cluster in control-CD45^{-}/control-CD45^{+}, Herts/33-CD45^{+}, and LaSota-CD45^{+}/CD45^{-}. G. Differences in pathway activities scored per cell by GSVA between myeloid cells isolated from different epithelial clusters, with enriched KO terms; $P < 0.05$. H and I.

Pseudotime trajectory plot representing the features of each cluster (H) and each sample (I) in the three identified states. K. Pie charts showing the proportion of cells in each sample in the three identified states.

Supplementary Figure 6. Single-cell profiling of T cells. A. Barplots showing the proportion of each sample in each cluster. B. Barplots showing the proportion of each cell type both *in vitro* and *in vivo*. C. Barplots showing the proportion of each cluster in each sample. D. t-SNE plot of the sample both *in vitro* and *in vivo*. E. t-SNE plot of the sample in control, Herts/33, LaSota, DF-1-control, and DF-1-Herts/33. F. t-SNE plot of the sample in control-$CD45^{+}/CD45^{-}$, Herts/33-$CD45^{+}/CD45^{-}$, LaSota-$CD45^{+}/CD45^{-}$, DF-1-control, and DF-1-Herts/33. G. t-SNE plot of each cell cluster in control, Herts/33, LaSota, DF-1-control, and DF-1-Herts/33. H. t-SNE plot of each cell cluster in control-$CD45^{+}/CD45^{-}$, Herts/33-$CD45^{+}/CD45^{-}$, LaSota-$CD45^{+}/CD45^{-}$, DF-1-control, and DF-1-Herts/33. I. Differences in pathway activities scored per cell by GSVA between T cells isolated from different T cell clusters, with enriched KO terms; $P < 0.05$. J–M. Pseudotime trajectory plot representing the features of each cluster *in vivo* (J), *in vitro* (K), and in each sample (L) in the three identified states. The five states were considered as three because states 3, 4, and 5 were on the same branch (M). N and O. Pie charts showing the proportion of cells in each sample in the three identified states.

Supplementary Figure 7. A. Only two cells with LaSota RNA were detected (UMI > 0) from total cells *in vivo*. B. Cells with LaSota RNA detected (UMI > 0) from fibroblast cells *in vivo*. C. Cells with LaSota RNA detected (UMI > 0) from cells *in vivo*. D. t-SNE plot of the virus genes of Herts/33 in each cluster. E. t-SNE plot of the virus genes of LaSota in each cluster. F–K. Proportion of virus$^+$ and virus$^-$ cells in different samples, and the composition of different samples in virus$^+$ and virus$^-$ cells.

Supplementary Figure 8. Cell–cell communication. Predicted number of interactions among mono-macro-neutrophil, T, fibroblast, endothelial, epithelial, unknown1, and unknown2 cells based on CellPhoneDB in the control, Herts/33, and LaSota groups.

Supplementary Table 1. Gene quantitative quality control overview of the sequencing library, alignment of scRNA-seq, and marker genes used for cell type identification.
